# Changes in ankylosing spondylitis incidence, prevalence and time to diagnosis over two decades

**DOI:** 10.1136/rmdopen-2021-001888

**Published:** 2021-12-09

**Authors:** Samantha S R Crossfield, Helena Marzo-Ortega, Sarah R Kingsbury, Mar Pujades-Rodriguez, Philip G Conaghan

**Affiliations:** 1 Leeds Institute for Data Analytics, University of Leeds, Leeds, UK; 2 Leeds Institute of Rheumatic and Musculoskeletal Medicine, University of Leeds, Leeds, UK; 3 NIHR Leeds Biomedical Research Centre, Leeds Teaching Hospitals NHS Trust, Leeds, UK; 4 Leeds Institute of Health Sciences, University of Leeds, Leeds, UK

**Keywords:** ankylosing spondylitis, spondylitis, ankylosing, epidemiology, arthritis

## Abstract

**Objectives:**

To assess changes in ankylosing spondylitis (AS) incidence, prevalence and time to diagnosis, between 1998 and 2017.

**Methods:**

Using UK GP data from the Clinical Practice Research Datalink, we identified patients diagnosed with AS between 1998 and 2017. We estimated the annual AS incidence, prevalence and length of time from first recorded symptom of back pain to rheumatology referral and diagnosis.

**Results:**

We identified 12 333 patients with AS. The incidence declined from 0.72 (±0.14) per 10 000 patient-years in 1998 to 0.39 (±0.06) in 2007, with this decline significant only in men, then incidence rose to 0.57 (±0.11) in 2017. By contrast, prevalence increased between 1998 and 2017 (from 0.13%±0.006 to 0.18%±0.006), rising steeply among women (from 0.06%±0.05 to 0.10%±0.06) and patients aged ≥60 (from 0.14%±0.01 to 0.26%±0.01). The overall median time from first symptom to rheumatology referral was 4.87 years (IQR=1.42–10.23). The median time from first symptom to diagnosis rose between 1998 and 2017 (from 3.62 years (IQR=1.14–7.07) to 8.31 (IQR=3.77–15.89)) and was longer in women (6.71 (IQR=2.30–12.36)) than men (5.65 (IQR=1.66–11.20)).

**Conclusion:**

AS incidence declined significantly between 1998 and 2007, with an increase between 2007 and 2017 that may be explained by an improvement in the recognition of AS or confidence in diagnosing AS over time, stemming from increased awareness of inflammatory back pain and the importance of early treatment. The rising AS prevalence may indicate improved patient survival. The persisting delay in rheumatology referral and diagnosis remains of concern, particularly in women.

**Key messages**

**What is already known about this subject?**

- Data on the incidence and prevalence of ankylosing spondylitis (AS) are sparse.
- There is a significant diagnostic delay in AS, which results in worse patient outcomes.

**What does this study add?**

- This population-based study, conducted in the UK across two decades and including 12 333 patients diagnosed with AS, showed a decrease in the incidence of AS among men and a rise in prevalence, especially in the over 60s.
- The observed lack of improvement in diagnostic delay over a 20-year period, appeared to be largely driven by delay in referral from primary care to rheumatology.

**How might this impact on clinical practice or further developments?**

- These results highlight the need to promote the recognition of inflammatory back pain among all health practitioners evaluating people with back pain, and to encourage referral of suspected AS cases to rheumatology.

## Introduction

Ankylosing spondylitis (AS) is a disabling chronic arthritis characterised by systemic inflammation leading to fusion of the axial skeleton, and represents the established phenotype within the axial spondyloarthritis (axSpA) disease spectrum. AS typically presents in a patient’s mid-20s and more commonly in men,^1 2^ causing significant morbidity in physical and mental health, with consequent impact on work productivity, at huge individual and societal cost.^3 4^

Estimates of the incidence and prevalence of AS range from 0.05 to 1.4/10 000 person-years and from 0.1% to 1.4%, respectively.^5^ In the UK, a national review highlighted that the incidence and prevalence of AS were uncertain.^6^

Treatment initiation in the earlier stages of disease associates with better clinical response and increased likelihood of remission.^7 8^ Unfortunately, timely diagnosis remains a challenge in the axSpA disease spectrum due to the lack of pathognomonic symptoms and signs of disease leading to significant delays between the first symptoms of inflammatory back pain (IBP) and the diagnosis being made.^9 10^ However, hospital-based studies may miss earlier reported symptoms of IBP that are often first presented in primary care, and studies of diagnostic delay tend not to distinguish AS from other concepts in axSpA.

Another important factor is the absence of diagnostic criteria for AS, despite the existence of diagnostic algorithms for axSpA.^11^ This has historically led to reliance on the presence of structural damage of the sacroiliac joints or spine for diagnosing AS, which presents late in the disease-course.^12^ Following the availability of effective treatment,^13^ there have been efforts to improve the recognition and diagnosis of AS in the earlier and non-radiographic disease stages. Classification criteria published in 2009 increased the sensitivity for recognising AS earlier and MRI has become an important tool for identifying inflammatory lesions before sacroiliitis is radiographically detectable.^14^ However, it is still uncertain whether this increased knowledge has contributed to a reduced diagnostic delay.

Using a large UK primary care dataset, we investigated trends in the incidence, prevalence and time to rheumatology referral and diagnosis in AS over two decades, to assess disease burden and the impact of modern practices in the diagnostic approach.

## Methods

This retrospective study is reported following current guidelines for observational studies (Strengthening the Reporting of Observational Studies in Epidemiology, online supplemental table 1).^15^ There was no patient-public involvement in the study, which used non-identifiable data, and dissemination of results to study participants is not possible.

10.1136/rmdopen-2021-001888.supp1 Supplementary data

### Data source

This study used the April 2018 update of the Clinical Practice Research Datalink (CPRD) GOLD dataset, which contains 17.6 million electronic health records from 734 UK general practitioner (GP) practices (approximately 8% of UK GP practices). The data source was established in 1987 and contains records that span the duration of a patient’s lifetime.^16^ CPRD data undergo quality assessment and patients have a comparable age, sex and ethnicity profile to the national census statistics and a body mass index distribution to the NHS Health Survey for England.^16–18^ The dataset has flags for patient records that are deemed to be of ‘acceptable’ quality for research and also contains the date from which the quality of the data from each contributing GP practice was deemed to be ‘up to standard’ (UTS).^16^ Coding quality is higher in data recorded while GP practices have UTS status.

### Study population

The eligible population had ≥1 day of registration during the study period (1 January 1998 to 1 April 2018) and excluded records flagged with ‘unacceptable’ research quality. Patient follow-up commenced from the latest of: the study start date, being aged ≥18 years, and having 1 year of UTS practice registration.^16^ Follow-up ended at the study end date, last data collection, practice deregistration, death or becoming aged ≥101 years.

The Read V.2 code, ‘N100.’ ‘Ankylosing spondylitis’, was used to define AS diagnoses and we excluded patients diagnosed before age 18 years. In a sensitivity analysis, we required an additional diagnostic or AS measurement code >7 days later, to improve diagnostic certainty (online supplemental table S2). The AS code N100. was previously validated on similar GP practice data (72% positive predictive value, PPV; 89% for two AS codes >7 days apart).^19^ The axSpA diagnosis code ‘N11F.’ was not used.

### Outcomes

The outcomes were the annual AS incidence and prevalence by sex, age and geographical area, and the annual time to diagnosis among women and men. Time to diagnosis was defined as the number of years between first coded non-specific back pain symptom and AS diagnosis.^20^ Time from first symptom to rheumatology referral, and from rheumatology referral to diagnosis, were secondary outcomes. The Read codes used for back pain symptoms and rheumatology referral are defined in online supplemental tables 3 and 4.

### Statistical analyses

We reported baseline patient characteristics and annual trends in outcomes (1 January 1998 to 31 December 2017). The sensitivity analysis ran until 31 December 2016 to enable >16 months of follow-up for the additional AS coding. The difference between calendar years was deemed significant where the 95% CIs did not overlap. All analyses were stratified by sex, except for years with ≤5 women or men.

**Figure 1:**
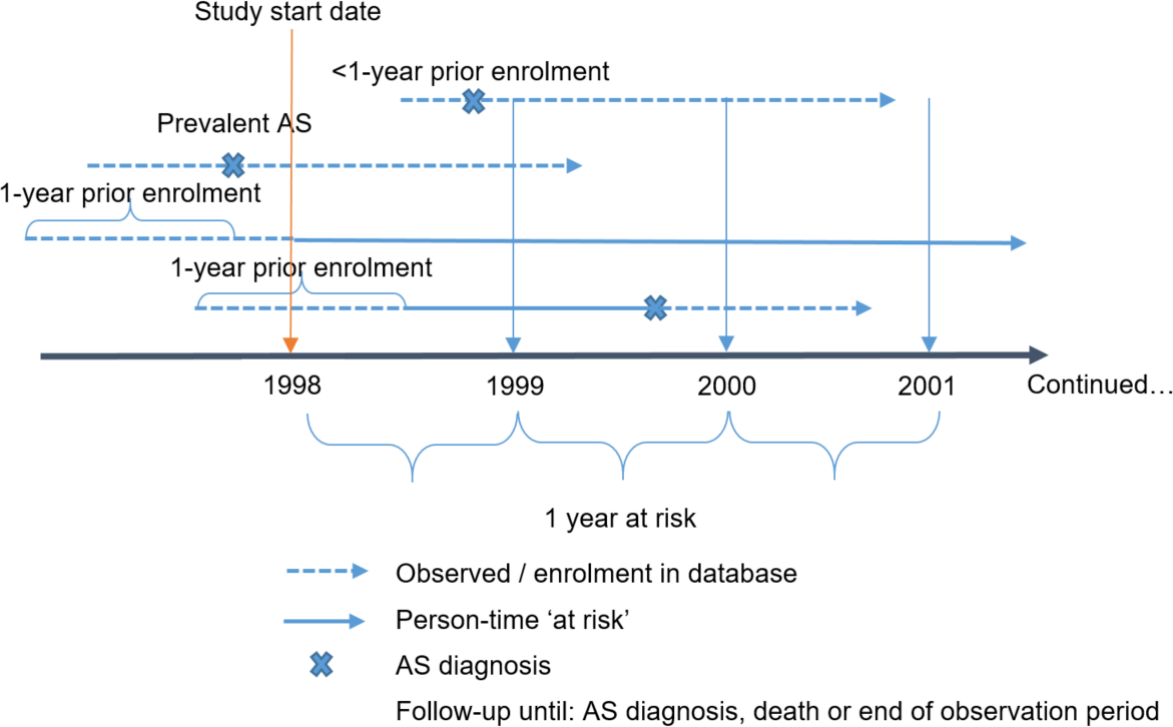
Study design for the ‘at risk’ cohort. AS diagnoses recorded during the period represented by a dashed line (observed/enrolment in database) contributed to prevalence but not incidence estimates. AS, ankylosing spondylitis.

Crude annual and period incidence rates per 10 000 person-years were calculated with 95% CIs for patients ‘at-risk’, that is having no AS diagnosis and ≥1 year of prior GP registration at the start of that time-period.^21^ This would exclude prevalent cases that might have been incorrectly recorded as incident diagnoses instead of medical history up to 1-year post-GP registration.^21^ Figure 1 shows the entry and ‘at-risk’ follow-up definitions for the incidence rate calculations. Crude point and period prevalence percentages were calculated with 95% CI, the former being calculated on 1 July of each calendar year. The annual percentage changes (APCs) and mean APC across 5-year intervals (1998–2002, 2003–2007, 2008–2012, 2012–2017) were calculated. The measures were stratified by sex, age (18–29 then 10-year bands until 90–99) and geography (where there was patient representation from ≥5 GP practices per region).^16^ Figures depicted age stratification in wider bands (18–29, 30–39, 40–59, 60–79, 80–99), for visual clarity.

The earliest recorded back pain symptom, and the first subsequent rheumatology referral, prior to AS diagnosis were identified. The median times between these were calculated overall and for patients diagnosed with AS in each year. Patients with ≥2 and ≥3 years of quality (UTS) registration prior to AS diagnosis were included in subanalyses of time from symptom to diagnosis.

R V.3.6.2, Microsoft SQL 2017 and Microsoft Excel 2016 were used.

## Results

### Cohort

**Figure 2:**
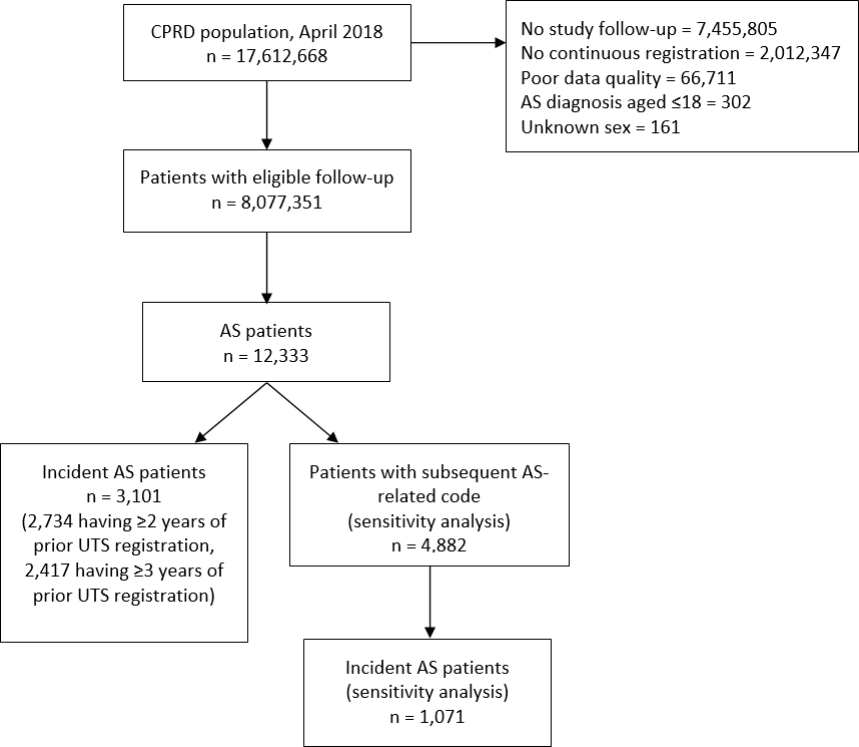
Study flow diagram of cohort selection. AS, ankylosing spondylitis; CPRD, Clinical Practice Research Datalink; UTS, up to standard.

A cohort of 12 333 patients with AS was identified (excluding 302 patients diagnosed aged ≤18), of whom 26.0% (N=3209) were women (figure 2). The median age at diagnosis was 36 (IQR=28–47) years and higher in women than men (online supplemental table S5). The median duration of follow-up per patient was 8.9 (IQR=3.6–14.2) years. There were 4882 patients with AS with a subsequent AS diagnostic or measurement code >7 days later, and these were included in sensitivity analyses. In the sensitivity analysis cohort, 22.4% (N=1095) were women and the median age at diagnosis was 34 (IQR=27–44) years. The distribution of socioeconomic deprivation was comparable between the primary and sensitivity analysis.

### Incidence

There were 3101 patients in the incident AS cohort (ie, AS diagnosed during follow-up), of whom 30.8% (N=951) were women. In the incident cohort, the median age at diagnosis was 43 (IQR=33–56) and the median duration of follow-up per patient was 12.6 (7.7–16.1) years. In sensitivity analyses (having a subsequent diagnostic or measurement code >7 days later), there were 1071 incident patients with AS, of whom 27.9% (N=299) were women and the median age at diagnosis was 40 (IQR=32–51) years.

The period incidence was 0.54 (±0.02) per 10 000 person-years; 0.19 (±0.01) in sensitivity analyses (online supplemental table S6). The at-risk person-years in each analysis were 57.5 million and 55.8 million (online supplemental figure S1). In both analyses, incidence was greatest among patients aged 30–39 (0.80±0.08 and 0.33±0.04, respectively) and 2.3 times higher in men (0.76±0.03 and 0.21±0.02, respectively) than women (0.33±0.02 and 0.09±0.06, respectively).

**Figure 3:**
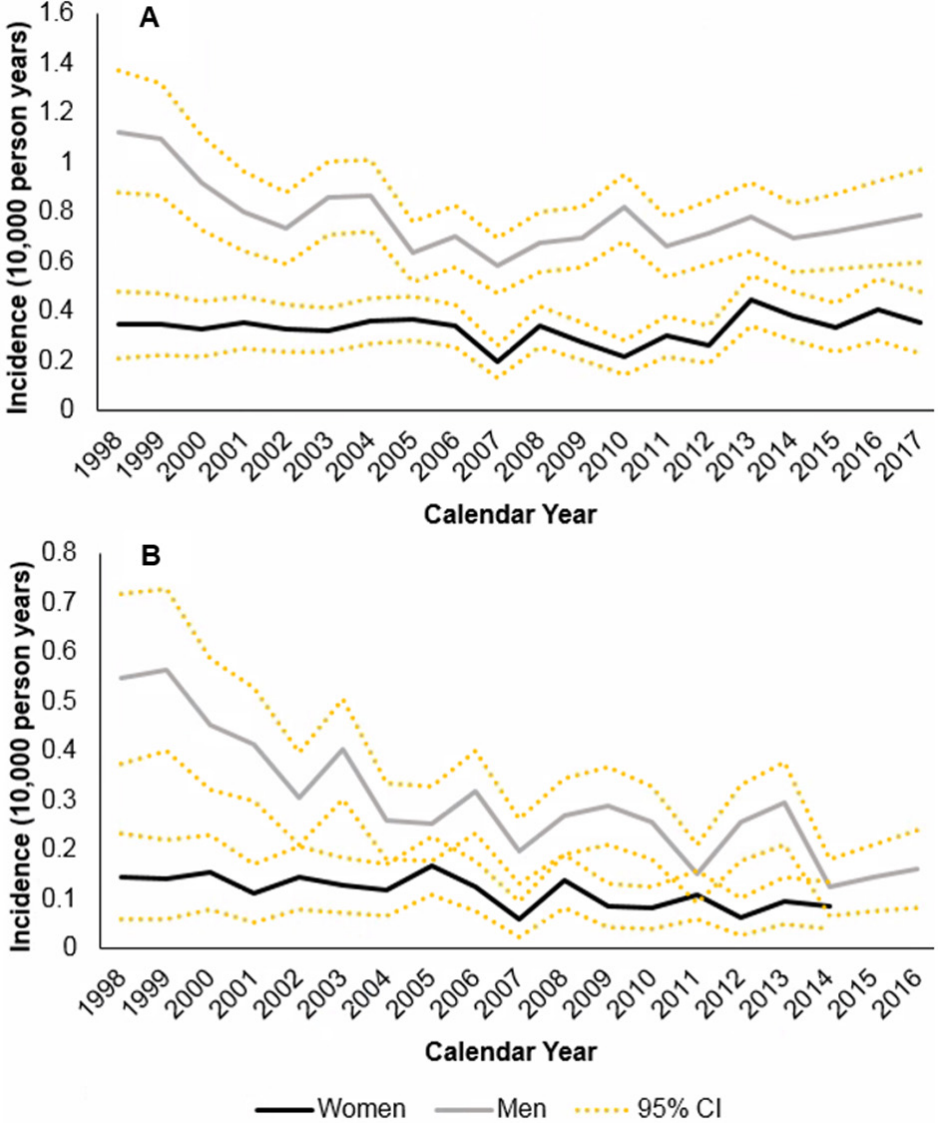
Annual incidence rate among women and men. Annual incidence rate among women and men, 1998–2017; (A) all patients (N=8 052 546); (B) patients in the sensitivity analysis (N=7 919 770).

Incidence declined significantly from 0.72 (±0.14) in 1998 to 0.39 (±0.06) in 2007, then rose significantly to 0.57 (±0.11) in 2017 (figure 3, online supplemental figure S2). The mean APC was −0.44: −7.42 in 1998–2002 and +3.72 in 2012–2017. The decline between 1998 and 2007 was significant in men but not women, and then both sexes showed a rising trend (online supplemental figure S3). Incidence was stable among patients aged ≥60 (online supplemental figure S4). In younger patients, incidence declined between 1998 and 2007 before rising. There was no clear regional trend (online supplemental figure S5).

**Figure 4:**
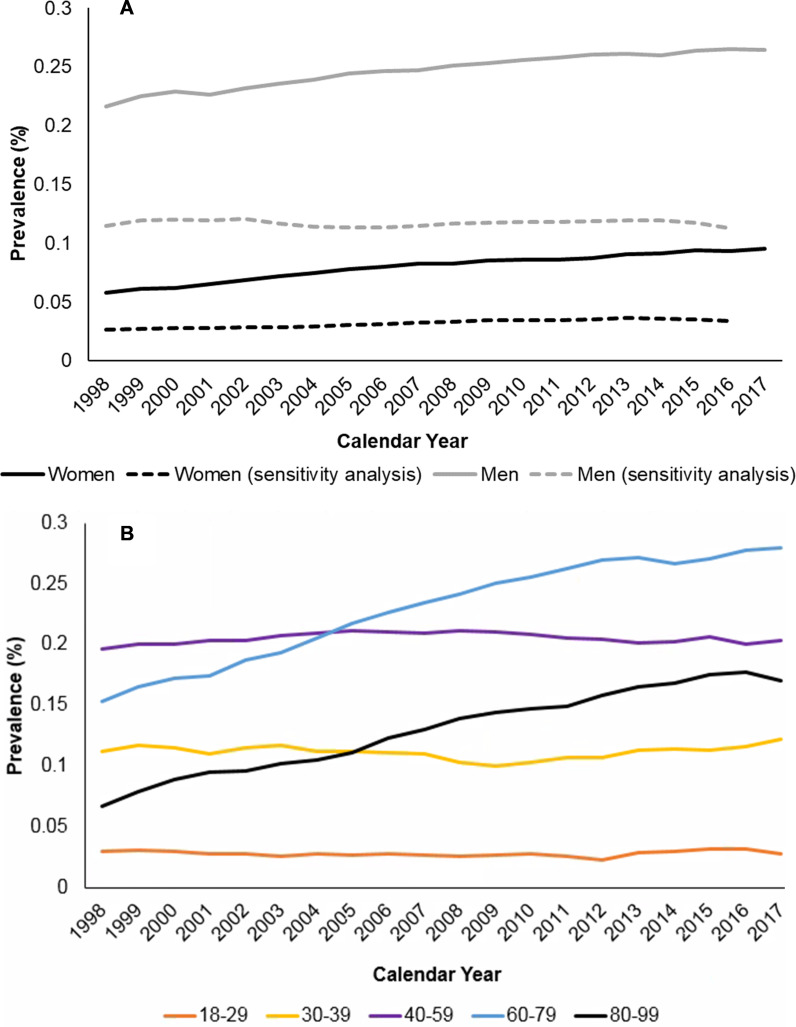
Annual percentage prevalence, by sex and by age-group. Annual percentage prevalence of ankylosing spondylitis (AS) (A) among women and men for all patients, 1998–2017 (N=8 052 980) and for patients in the sensitivity analysis, 1998–2016 (N=7 413 674); (B) per age-group among patients aged 18–99, 1997–2017 (N=7 532 700). Sensitivity analysis=patients with an additional diagnostic or AS measurement code recorded >7 days later.

**Figure 5:**
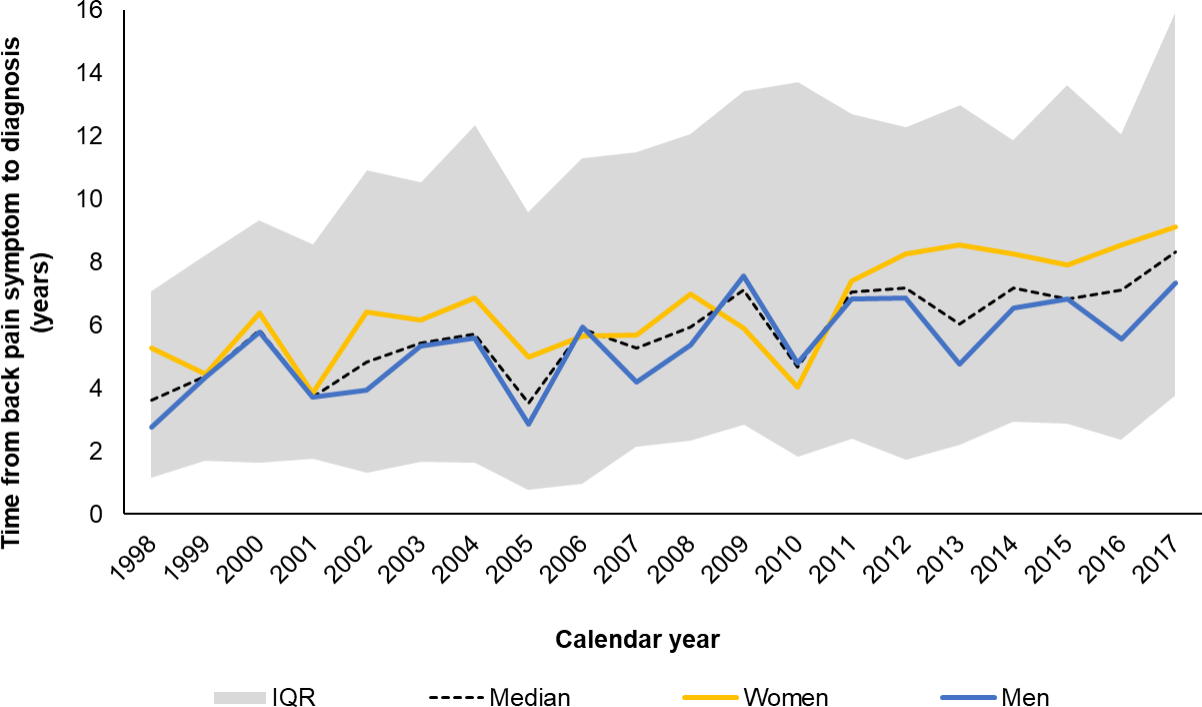
Annual median time in years from first recorded symptom to diagnosis. Annual median time in years from first recorded symptom to diagnosis, with IQR, 1998–2017 (N=2120). Dashed line represents the overall annual diagnostic delay, and the shaded area the IQR.

In sensitivity analyses, incidence was 0.34 (±0.09) in 1998 and declined more steeply to 0.13 (±0.04) in 2007, but then stabilised (0.10±0.04 in 2016). The mean APC was −2.91: −9.89 in 1998–2002 and +1.07 in 2012–2016. The initial decline was significant in both sexes, with no significant change after 2007 (online supplemental figure S6). Incidence was stable among patients aged ≥60 (online supplemental figure S7). In younger patients, incidence declined between 1998 and 2007 before stabilising. There was no clear regional trend (data not shown).

### Prevalence

The period prevalence of AS was 0.15% (±0.003) (online supplemental table S7). Prevalence was greatest among patients aged 60–69 (0.26% ±0.01) and 2.9 times higher in men (0.23% ±0.01) than women (0.08% ±0.003). Prevalence was lowest in London (0.11% ±0.01). In sensitivity analyses, the period prevalence was 0.06% (±0.002) and the demographic and regional patterns were consistent with the main analysis.

Prevalence rose significantly from 0.13% (±0.01) in 1997 to 0.18% (±0.01) in 2017; more steeply in women than men (mean APC +2.69 and +1.07) (figure 4). Annual prevalence was stable in patients aged <60, and rose in older cohorts, notably in patients aged 70–79 (0.13% ±0.02 in 1998, 0.30% ±0.02 in 2017). There was little regional variation over time.

The annual prevalence was stable in sensitivity analyses (0.07% ±0.004 in 1998 and 2016), although there was a rising trend in women (mean APC +1.43). The annual prevalence declined in patients aged <60, and rose in older cohorts, notably in patients aged 70–79 (online supplemental figure S8). There was little regional variation over time.

At AS diagnosis, 2120 (68.4%) patients had a prior-recorded back-pain symptom. The proportion with a prior-recorded back-pain symptom rose between 1998 and 2017 (from 60.4% to 77.6%) and was higher in women (70.6%) than in men (67.4%) (online supplemental figures S9 and S10). Results from sensitivity analyses and in UTS-related subanalyses were consistent (online supplemental figures S11 and S12).

The median time from first back pain symptom to diagnosis was 6.0 years (IQR=1.9–11.6), higher in women (6.7, 2.3–12.4) than men (5.7, 1.7–11.2) and in subanalyses with ≥2 and ≥3 quality registration years (6.2, 2.1–11.8 and 6.6, 2.4–12.0, respectively). In sensitivity analyses, the time to diagnosis was lower (5.2 years, IQR=1.7–10.9), again higher in women (6.0, 2.4–12.0) than men (4.9, 1.4–10.4) and higher in UTS-related subanalyses (5.5, 1.9–11.2; 6.0, 2.2–11.3).

During the study period, time to diagnosis more than doubled from 3.6 (IQR=1.1–7.1) years (5.3, 1.7–6.9 in women; 2.7, 1.1–7.1 in men) in 1998 to 8.3 (3.7–15.9) years (9.1, 5.6–12.3 in women, 7.3, 2.0–16.4 in men) in 2017 (figure 5). In sensitivity analyses, the time to diagnosis was lower (5.2 years, IQR=1.7–10.9) with less clear change, again higher in women (online supplemental figures S13 and S14). The UTS-related subanalyses for both showed consistent trends although the time to diagnosis was higher (online supplemental figures S15 and S16).

The recording of rheumatology referrals increased over time (online supplemental figures S17 and S18). The median time from symptom onset to referral was 4.9 years (IQR=1.4–10.2; N=819), higher in women (5.2, 1.8–11.1) than men (4.5, 1.2–9.7). Time to referral more than doubled between 1998 and 2017 (from 2.2, IQR=0.8–4.0 to 5.7, 2.0–10.2) (online supplemental figure S19). The median time from referral to diagnosis was 0.6 years (IQR=0.1–2.7; N=1167), two times higher in women (1.0 (0.2–3.2)) than men (0.5 (0.1–2.6)) and with no clear change over time (online supplemental figure S20). In sensitivity analyses (having a subsequent diagnostic or measurement code >7 days later), the patterns were consistent (online supplemental figure S21).

## Discussion

In this first study of AS incidence and prevalence in the UK, incidence appeared to decline between 1998 and 2007. From 2007 to 2017, there was an apparent rise in incidence that was not seen in the sensitivity analysis. Prevalence rose among patients aged ≥60 and among women. There was a trend to worsening in delay to referral and diagnosis over two decades, particularly in women, with no reduction in the sex difference.

### Incidence and prevalence

From 2007 to 2017, an increasing proportion of patients had a single (rather than ≥2 instances of) AS code during follow-up (ie, were excluded from sensitivity analyses). This may suggest increased awareness of AS, in cases where it indicates that AS was suspected but not confirmed. British guidelines published in 2005 on treating AS with biologicals may have raised awareness and prompted investigation of suspected cases of AS.^13^ This would suggest that incident and prevalent estimates of AS from sensitivity analyses post-2007 may be most accurate. Interestingly, an increase in single AS coding in women was also reported following the introduction of biologicals in Canada.^22^ An increasing proportion with a single code may also reflect the increasing referral to rheumatology that we noted, which may also reflect increased awareness of the need to treat AS with biologicals. Following referral, confirmed cases of AS may be treated exclusively in rheumatology, with no further coding in GP records as a result. Alternatively, recording of AS diagnoses through single but not multiple coding events may have improved over time, which would again suggest that more recent estimates of incidence and prevalence might be more accurate. One consideration is whether there may have been increasing use of the axSpA diagnosis following the publication of the Assessment of SpondyloArthritis International Society (ASAS) classification criteria in 2009.^14^ However, this study showed no change in the incidence and prevalence of AS from 2009, suggesting that the evolving nomenclature may have had limited effect in primary care coding of AS. Further, the axSpA code only first appeared in the CPRD GOLD in 2015 and this study found no change in incidence and prevalence from 2015.

The period prevalence in our study was comparable to the weighted mean prevalence of 0.19% estimated from other European countries.^23^ While mortality is higher in AS than in the general population,^24^ our reported rising prevalence may suggest improved survival in more recent years. A hospital-based study of AS in Norway had found comparable survival between AS and non-AS women.^25^

### Time to diagnosis

It is important to note that we found no improvement in the diagnostic delay of AS since this was first highlighted in 1999,^26^ and UTS-related subanalyses suggest delay might be even greater. The commonality of chronic back pain^27^ and the complexity of AS, with diverse symptoms,^28^ may continue to impede early diagnosis. The sex difference in diagnostic delay of a year was greater than the 7-month difference reported in a systematic review of delay in all spondyloarthritides.^29^ The reported increase in the duration of delay corresponds with survey reports of 6-year and 8.5-year delay in 2010 and 2016, respectively in the UK.^10 30^ The apparent delay to referral highlights the necessity of raising awareness of IBP and the associated features of AS among non-rheumatologists. Indeed, a survey of GPs reported only 13% and 50%, respectively recognised alternating buttock pain and pain improving with exercise as suggestive of IBP, and 60% recognised uveitis as an associated AS feature.^31^ Encouragingly, UK guidelines published subsequently in 2019 have highlighted the importance of early referral to rheumatology in AS.^28^ However, the delay from referral to diagnosis that we also found, which is twice as long in women as in men, also requires investigation. This delay suggests that some of the difficulties in diagnosing AS apply to rheumatologists also, as was reported in a survey of secondary care specialists.^32^

A positive finding, however, is that the recording of back pain symptoms increased over time. This suggests that while a survey published in 2011 found that almost half of patients with AS waited over a year after developing symptoms before contacting a healthcare professional,^30^ patients might be making contact earlier or more often in more recent years. If the ‘pre-contact’ disease duration has shortened over time, the more recent measures of the time to diagnosis in this study may more accurately reflect the diagnostic delay. The rising recording of back pain symptoms over time may also indicate that data quality has improved over time, which would again indicate that the more recent estimates of time to diagnosis are more accurate.

The increase in the recording of referrals over time suggests increasing awareness among GPs of AS symptoms and the importance of referral for rheumatology-led biological therapy.^13^ The apparent rise in referral is corroborated by survey reports that show the proportion of patients with AS currently attending a rheumatology clinic rose from 68% in 2010 to 82% in 2016.^10 30^

### Strengths and limitations

Study strengths included long-term follow-up (median 12.6 years) of a large population-based cohort, which is important for the identification of early symptoms and rheumatology referrals recorded in the years prior to the diagnosis of AS. We explored AS in primary care where symptoms first present, potentially capturing early presentations of back pain symptoms and ensuring that GP-diagnosed cases were not missed.^30^ Further, the code used to define AS diagnosis had previously been validated on GP practice data (72% PPV)^19^ and the robustness of the diagnostic certainty in this study was confirmed by the comparability of results between primary and sensitivity analyses (using an additional code >7 days apart). For measuring diagnostic delay, the UTS-related subanalyses revealed the importance of long-term quality follow-up for capturing earlier symptom presentations. Increased symptom recording over time will have improved the accuracy of more recent measures of diagnostic delay.

Study limitations include those common to routine data-based studies, for example, incomplete and changing coding practices.^33^ AS diagnoses were not clinically validated as part of this study, however the AS code N100. was previously validated on GP practice data (72%, PPV; 89% for two AS codes >7 days apart).^19^ Importantly, spondyloarthritis nomenclature evolved during the study time-frame, leading to an increasing use of the concept of axSpA among rheumatologists. However, it appears that this evolving nomenclature may predominantly affect secondary care, as we found no change in AS recording by GPs after the ASAS classification criteria were published in 2009.^14^ In corroboration, the axSpA Read code only first appeared in the CPRD GOLD in 2015 and we found no change in results from 2015. Indeed, the axSpA code was recorded in only 201 clinical events (compared with 1203 for the AS code) by the time of data extract for this study (ie, between 2015 and 2018), and so inclusion of the code would have had little impact on results in this study. Recording of rheumatology referrals increased over time but it was not possible to determine whether referrals occurred without this being recorded using Read codes. However, surveys from 2010 and 2016 reported 68% and 82% of patients respectively being under the care of a rheumatologist, and in our study 49% and 60% had a rheumatology referral prior to AS diagnosis in 2010 and 2016; the comparability suggests a high level of referral recording, given the ongoing role of rheumatology in AS management, postdiagnosis.^10 30^ A survey by Hamilton *et al* reported that, over time in the UK, diagnoses of AS have increasingly been made in the specialist rheumatology set up rather than primary care.^30^ This may in part explain the apparent increase in diagnostic delay found in this study over time, as a temporal lag in the recording of rheumatology-led diagnoses in GP records is possible, although our data show that the time from rheumatology referral to AS diagnosis contributed only marginally to the overall diagnostic delay. It is also possible that some diagnoses reported to GPs from secondary care may not be recorded in GP records other than as a scanned letter, though this may be more likely to affect acute than chronic diseases, or that diagnoses may be misclassified. Linkage between rheumatology and GP practice data would be required in order to investigate completeness and its impact on the study. Back pain symptom recording increased over time, which suggests that the information on symptoms may be more complete in recent years, though back pain symptom codes exist in the dataset back to the 1940s. Unfortunately, we could not explore delayed presentation of symptoms to primary care. This is another significant factor contributing to diagnostic delay, which has been reported using survey data,^30^ which suggests the importance of raising awareness of AS not only among health professionals but in the general population.

As characterises open cohort observational studies, we used a dataset with a changing population structure and varying follow-up duration. However, we designed our investigation to account for this, including by reporting incidence with the denominator in person-years from an at-risk cohort and point prevalence rather than annual period prevalence.^34 35^ Further, the number of incident diagnoses per year (106 in 1998, 98 in 2017) fluctuated but remained sufficient to perform the analyses. Finally, while this study used UK data, the considerations for improving the education and recognition of IBP and development of a care pathway are universal, with other countries reporting similar challenges with symptom recognition and inconsistent approaches to diagnostic investigation and management in AS and axSpA.^36^

### Conclusion

In conclusion, the results of the primary and sensitivity analysis suggest that between 2007 and 2017 the incidence of AS may have stabilised or increased and awareness may have improved among GPs in the UK. The rising prevalence in the over 60s may indicate improved survival and highlights the importance of appropriate AS management in an ageing population. Unfortunately, the delay to diagnosis particularly in women has persisted over 20 years and appears to be largely driven by delay in referral to rheumatology. This delay highlights the need for education and increased awareness of IBP and associated AS features among non-rheumatologists.

## Data Availability

Data may be obtained from a third party and are not publicly available. The CPRD data were provided under a licence that does not permit sharing. The code-lists used in definitions and the derived results are published in the manuscript and supporting file.
