# Supplementary material for: Changes in ankylosing spondylitis incidence, prevalence and time to diagnosis over two decades
Source: RMD Open. 2021 Dec 9;7(3):e001888. doi: 10.1136/rmdopen-2021-001888 (PMC8663075; doi:10.1136/rmdopen-2021-001888)
Supplement: Supplementary data [file rmdopen-2021-001888supp001.pdf]

# Supplementary Material

## Table of Contents


Glossary of Terms ........ 2

Supplementary Methods ........ 3

Supplementary Table S1. STROBE Checklist for cohort studies with reference to this study ........ 3

Supplementary Table S2. Read Codes used to confirm the initial AS diagnosis, in the sensitivity analysis ........ 5

Supplementary Table S3. Read Codes used to determine back pain ........ 5

Supplementary Table S4. Read Codes used to indicate a rheumatology referral had occurred ........ 5

Supplementary Results ........ 6

Supplementary Table S5. Number of AS patients in the primary and sensitivity analysis, overall and diagnosed during follow-up, and median age at diagnosis, by sex ........ 6

Supplementary Table S6. Incidence of AS by calendar year and stratified by sex, age-group and geographical area (N = 7,532,147) ........ 7

Supplementary Table S7. Percentage prevalence of AS by calendar year and stratified by sex, age-group and geographical area (N = 7,532,980) ........ 8

Supplementary Figure S1. Person-years (million) in the incidence 'at-risk' cohort per year, 1998-2017 (N = 8,052,546) ........ 9

Supplementary Figure S2. Annual incidence rate of AS defined as having ≥1 AS diagnostic code, 1998-2017 (N = 8,052,546), and in the sensitivity analysis, 1998-2016 (N = 7,919,770) ........ 9

Supplementary Figure S3. Annual incidence rate of AS in women and men, 1998-2017 (N = 8,052,546) ........ 10

Supplementary Figure S4. Annual incidence rate of AS by age-group, 1998-2017 (N = 8,051,097) ........ 10

Supplementary Figure S6. Annual incidence rate of AS in women and men in the sensitivity analysis, 1998-2017 (N = 7,919,770) ........ 11

Supplementary Figure S7. Annual incidence rate of AS in the sensitivity analysis by age-group, 1998-2017 (N = 7,918,339) ........ 12

Supplementary Figure S8. Annual percentage prevalence of AS per age-group among patients aged 18-99 in the sensitivity analysis, 1997-2016 (N = 7,506,959) ........ 12

Supplementary Figure S9. Annual percentage of patients diagnosed with AS (having ≥1, ≥2 and ≥3 years of prior UTS registration) that had a prior back-pain symptom code, 1998-2017 (N = 3,101; N = 2,734; N = 2,417) ........ 13




Crossfield SSR, *et al. RMD Open* 2021; 7:e001888. doi: 10.1136/rmdopen-2021-001888

Supplementary Figure S10. Annual percentage of patients diagnosed with AS (having ≥1, ≥2 and ≥3 prior years of UTS) that had a prior back-pain symptom code, for women and men 1998-2017 (N = 3,101; N = 2,734; N = 2,417) ..... 13

Supplementary Figure S11. Annual percentage of patients with in the sensitivity analysis, having ≥1, ≥2 and ≥3 prior years of UTS, who had a prior back-pain symptom code, 1998-2016 (N = 1,071; N = 957; N = 821) ..... 14

Supplementary Figure S12. Annual percentage of patients in the sensitivity analysis, having ≥1, ≥2 and ≥3 prior years of UTS, who had a prior back-pain symptom code, for women and men, 1998-2016 (N = 1,071; N = 957; N = 821) ..... 15

Supplementary Figure S13. Median time in years from first recorded symptom to first diagnosis in the sensitivity analysis, 1998-2016 (N = 757) ..... 16

Supplementary Figure S14. Median time in years from first recorded symptom to first diagnosis in the sensitivity analysis, for women and men, 1998-2016 (N = 757) ..... 16

Supplementary Figure S16. Median time in years from symptom to diagnosis, for patients with ≥1, ≥2 and ≥3 years prior UTS registration in the sensitivity analysis, 1998-2016 (N = 757; N = 688; N = 606) ..... 17

Supplementary Figure S17. Annual percentage of patients diagnosed with AS that had prior back pain and rheumatology referral, for women and men 1998-2017 (N = 3,101) ..... 18

Supplementary Figure S18. Annual percentage of patients diagnosed with AS that had a prior rheumatology referral, for women and men 1998-2017 (N = 3,101) ..... 18

Supplementary Figure S19. Annual median time in years from first recorded back pain to rheumatology referral, for men and women, 1998-2017 (N = 819) ..... 19

Supplementary Figure S20. Annual median time in years from first rheumatology referral to diagnosis, for women and men: A) primary analysis, 1998-2017 (N = 1,167); B: sensitivity analysis, 1998-2016 (N = 399) ..... 20

Supplementary Figure S21. Annual median time in years from first recorded back pain symptom to rheumatology referral, for men and women in the sensitivity analysis, 1998-2016 (N = 279) ..... 21

## Glossary of Terms

| | |
|---|---|
| CPRD | The Clinical Research Practice Datalink |
| AS | Ankylosing spondylitis |
| STROBE | Strengthening the Reporting of Observational Studies in Epidemiology |

## Supplementary Methods

**Supplementary Table S1. STROBE Checklist for cohort studies with reference to this study**

| | Item No | Recommendation | Page No |
|---|---|---|---|
| **Title and abstract** | 1 | (*a*) Indicate the study's design with a commonly used term in the title or the abstract | 2 |
| | | (*b*) Provide in the abstract an informative and balanced summary of what was done and what was found | 2 |
| **Introduction** | | | |
| Background/rationale | 2 | Explain the scientific background and rationale for the investigation being reported | 5 |
| Objectives | 3 | State specific objectives, including any prespecified hypotheses | 6 |
| **Methods** | | | |
| Study design | 4 | Present key elements of study design early in the paper | 6 |
| Setting | 5 | Describe the setting, locations, and relevant dates, including periods of recruitment, exposure, follow-up, and data collection | 6, 7 |
| Participants | 6 | (*a*) Give the eligibility criteria, and the sources and methods of selection of participants. Describe methods of follow-up | 6, 7 |
| | | (*b*) For matched studies, give matching criteria and number of exposed and unexposed | NA |
| Variables | 7 | Clearly define all outcomes, exposures, predictors, potential confounders, and effect modifiers. Give diagnostic criteria, if applicable | 7 |
| Data sources/ measurement | 8* | For each variable of interest, give sources of data and details of methods of assessment (measurement). Describe comparability of assessment methods if there is more than one group | 7, 8 |
| Bias | 9 | Describe any efforts to address potential sources of bias | 7, 8 |
| Study size | 10 | Explain how the study size was arrived at | 6, 7 |
| Quantitative variables | 11 | Explain how quantitative variables were handled in the analyses. If applicable, describe which groupings were chosen and why | 7, Sup. Material |
| Statistical methods | 12 | (*a*) Describe all statistical methods, including those used to control for confounding | 7, 8 |
| | | (*b*) Describe any methods used to examine subgroups and interactions | 7, 8 |
| | | (*c*) Explain how missing data were addressed | NA |
| | | (*d*) If applicable, explain how loss to follow-up was addressed | NA |
| | | (*e*) Describe any sensitivity analyses | 7 |
| **Results** | | | |
| Participants | 13* | (a) Report numbers of individuals at each stage of study—eg numbers potentially eligible, examined for | 8, 9, Fig. 2 |



Crossfield SSR, *et al. RMD Open* 2021; 7:e001888. doi: 10.1136/rmdopen-2021-001888

| | | | |
|---|---|---|---|
| | | eligibility, confirmed eligible, included in the study, completing follow-up, and analysed | |
| | | (b) Give reasons for non-participation at each stage | 8, Fig. 2 |
| | | (c) Consider use of a flow diagram | 8, 9 Fig. 2 |
| Descriptive data | 14* | (a) Give characteristics of study participants (eg demographic, clinical, social) and information on exposures and potential confounders | 8, 9, Sup. Table S5 |
| | | (b) Indicate number of participants with missing data for each variable of interest | NA |
| | | (c) Summarise follow-up time (eg, average and total amount) | 8, 9, Sup. Table S6 |
| Outcome data | 15* | Report numbers of outcome events or summary measures over time | 8-11, Sup. Tables S6, S7 |
| Main results | 16 | (*a*) Give unadjusted estimates and, if applicable, confounder-adjusted estimates and their precision (eg, 95% confidence interval). Make clear which confounders were adjusted for and why they were included | 9-11 |
| | | (*b*) Report category boundaries when continuous variables were categorized | 8, Sup. Material |
| | | (*c*) If relevant, consider translating estimates of relative risk into absolute risk for a meaningful time period | NA |
| Other analyses | 17 | Report other analyses done—eg analyses of subgroups and interactions, and sensitivity analyses | 9-11 |
| **Discussion** | | | |
| Key results | 18 | Summarise key results with reference to study objectives | 11 |
| Limitations | 19 | Discuss limitations of the study, taking into account sources of potential bias or imprecision. Discuss both direction and magnitude of any potential bias | 14, 15 |
| Interpretation | 20 | Give a cautious overall interpretation of results considering objectives, limitations, multiplicity of analyses, results from similar studies, and other relevant evidence | 15 |
| Generalisability | 21 | Discuss the generalisability (external validity) of the study results | 14, 15 |
| **Other information** | | | |
| Funding | 22 | Give the source of funding and the role of the funders for the present study and, if applicable, for the original study on which the present article is based | 17 |

**Supplementary Table S2. Read Codes used to confirm the initial AS diagnosis, in the sensitivity analysis**

| Code | Term Description |
|---|---|
| N100. | Ankylosing spondylitis |
| 2377. | ankyl.spondyl.chest def. |
| 388p. | BASDAI - Bath ankylosing spondylitis disease activity index |
| 388p0 | Bath Ankylosing Spondylitis Functional Index |
| 38QL. | Bath Ankylosing Spondylitis Metrology Index |

**Supplementary Table S3. Read Codes used to determine back pain**

| Code | Term Description |
|---|---|
| N142. | Low back pain |
| 16C9. | Chronic low back pain |
| 16CA. | Mechanical low back pain |
| 16C6. | Back pain without radiation NOS |
| N149. | Back stiffness |
| N10z. | Spondylitis NOS |
| 16C7. | C/O - upper back ache |
| 16C.. | Backache symptom |
| 16C2. | Backache |
| 16C3. | Backache with radiation |
| 16C8. | Exacerbation of backache |
| 16CZ. | Backache symptom NOS |
| 14G4. | H/O: back problem |
| N145. | Backache; unspecified |
| N141. | Pain in thoracic spine |
| 16C5. | C/O - low back pain |
| N1460 | Lumbosacral ankylosis |
| N1461 | Sacroiliac ankylosis |
| N1462 | Sacral ankylosis NOS |
| N1466 | Sacroiliac disorder |
| N148. | Ankylosis/instability of cervical;thoracic or lumbar spine |
| N1486 | Lumbar spine ankylosis |
| N14z. | Ankylosis of spine NOS |

**Supplementary Table S4. Read Codes used to indicate a rheumatology referral had occurred**

| Code | Term Description |
|---|---|
| 66H9. | Rheumatology management plan given |
| 67Ih. | Advice to GP from rheumatology service |
| 8H2C. | Admit rheumatology emergency |
| 8H3H. | Non-urgent rheumatology admisn |
| 8H4B. | Referred to rheumatologist |
| 8HJC. | Rheumatology self-referral |
| 8HKA. | Rheumatology D.V. requested |
| 8HLA. | Rheumatology D.V. done |
| 8HMA. | Listed for Rheumatology admiss |
| 8HTd. | Referral to rheumatology clinic |
| 8HTP. | Referral to musculoskeletal clinic |
| 8HVQ. | Private referral to rheumatologist |

| | |
|---|---|
| 99HB. | Rheumatology disorder annual review |
| 9N0w. | Seen in musculoskeletal clinic |
| 9N1C0 | Rheumatology service home visit |
| 9N1O. | Seen in rheumatology clinic |
| 9NIR. | Seen by rheumatology nurse specialist |
| 9NNT. | Under care of rheumatologist |
| ZL18T | Under care of rheumatologist |
| ZL22G | Under care of rheumatology nurse specialist |
| ZL5AR | Referral to rheumatologist |
| ZL62G | Referral to rheumatology nurse specialist |
| ZL9AT | Seen by rheumatologist |
| ZLA2G | Seen by rheumatology nurse specialist |
| ZLD3T | Discharge by rheumatologist |
| ZLD7E | Discharge by rheumatology nurse specialist |
| ZLE6Q | Discharge from rheumatology service |

## Supplementary Results

**Supplementary Table S5. Number of AS patients in the primary and sensitivity analysis, overall and diagnosed during follow-up, and median age at diagnosis, by sex and Index of Multiple Deprivation (2015) quintile**

| | AS in primary analysis | | AS in sensitivity analysis | |
|---|---|---|---|---|
| | Full cohort | Incident cohort | Full cohort | Incident cohort |
| Patient count (%) | 12,333 | 3,101 | 4,882 | 1,071 |
| Women | 3,209 (26.0) | 953 (30.8) | 1,095 (22.4) | 299 (27.9) |
| Men | 9,124 (74.0) | 2,148 (69.2) | 3,787 (77.6) | 772 (72.1) |
| Median age at diagnosis (IQR) | 36 (28-47) | 43 (33-56) | 34 (27-44) | 40 (32-51) |
| Women | 38 (30-50) | 43 (33-55) | 35 (28-46) | 40 (33-51) |
| Men | 35 (28-46) | 43 (33-56) | 34 (27-43) | 40 (31-50) |
| Index of Multiple Deprivation quintile (%¥) | 7,276 | 1,751 | 2,970 | 611 |
| 1 (least deprived) | 1,789 (24.6) | 429 (24.5) | 723 (24.3) | 163 (26.7) |
| 2 | 1,715 (23.6) | 395 (22.6) | 699 (23.5) | 121 (19.8) |
| 3 | 1,553 (21.3) | 368 (21.0) | 644 (21.7) | 119 (19.5) |
| 4 | 1,243 (17.1) | 300 (17.1) | 512 (17.2) | 114 (18.7) |
| 5 (most deprived) | 976 (13.4) | 259 (14.8) | 392 (13.2) | 94 (15.4) |

Note: AS = ankylosing spondylitis, IQR = interquartile range
¥Percentage calculated for participants with available data.



Crossfield SSR, *et al. RMD Open* 2021; 7:e001888. doi: 10.1136/rmdopen-2021-001888

**Supplementary Table S6. Incidence of AS by calendar year and stratified by sex, age-group and geographical area (N = 7,532,147)**

| | Number of events (person-years at risk / 10,000) | | Incidence rate per 10,000 person-years (95% CI) | |
|---|---|---|---|---|
| | Main analysis | Sensitivity analysis | Main analysis | Sensitivity analysis |
| Overall | 3,102 (5,753.9) | 1,071 (5,585.9) | 0.54 (±0.02) | 0.19 (±0.01) |
| *Year* | | | | |
| 1998 | 106 (146.3) | 50 (146.4) | 0.72 (±0.14) | 0.34 (±0.09) |
| 1999 | 119 (166.9) | 58 (167.0) | 0.71 (±0.13) | 0.35 (±0.09) |
| 2000 | 125 (202.6) | 61 (202.8) | 0.62 (±0.11) | 0.30 (±0.08) |
| 2001 | 142 (246.3) | 64 (246.5) | 0.58 (±0.09) | 0.26 (±0.06) |
| 2002 | 145 (273.5) | 61 (273.7) | 0.53 (±0.09) | 0.22 (±0.06) |
| 2003 | 181 (307.7) | 81 (308.0) | 0.59 (±0.09) | 0.26 (±0.06) |
| 2004 | 202 (329.6) | 62 (329.9) | 0.61 (±0.08) | 0.19 (±0.05) |
| 2005 | 177 (351.6) | 74 (351.9) | 0.50 (±0.07) | 0.21 (±0.05) |
| 2006 | 186 (357.5) | 79 (357.8) | 0.52 (±0.07) | 0.22 (±0.05) |
| 2007 | 140 (359.3) | 46 (359.6) | 0.39 (±0.06) | 0.13 (±0.04) |
| 2008 | 183 (361.1) | 73 (361.4) | 0.51 (±0.07) | 0.20 (±0.05) |
| 2009 | 176 (361.6) | 68 (362.0) | 0.49 (±0.07) | 0.19 (±0.04) |
| 2010 | 183 (356.0) | 60 (356.3) | 0.51 (±0.07) | 0.17 (±0.04) |
| 2011 | 166 (346.7) | 45 (347.0) | 0.48 (±0.07) | 0.13 (±0.04) |
| 2012 | 167 (341.1) | 54 (341.5) | 0.49 (±0.07) | 0.16 (±0.04) |
| 2013 | 198 (323.9) | 63 (324.2) | 0.61 (±0.09) | 0.19 (±0.05) |
| 2014 | 158 (293.8) | 31 (294.1) | 0.54 (±0.08) | 0.11 (±0.04) |
| 2015 | 133 (253.2) | 20 (253.5) | 0.53 (±0.08) | 0.08 (±0.03) |
| 2016 | 117 (202.3) | 21 (202.5) | 0.58 (±0.10) | 0.10 (±0.04) |
| 2017 | 98 (172.9) | | 0.57 (±0.11) | |
| *Sex* | | | | |
| Female | 954 (2,919.6) | 299 (2,833.3) | 0.33 (±0.02) | 0.09 (±0.01) |
| Male | 2,148 (2,834.3) | 772 (2,752.6) | 0.76 (±0.03) | 0.21 (±0.02) |
| *Age-group* | | | | |
| 18-19 | 31 (115.7) | 11 (112.2) | 0.27 (±0.09) | 0.09 (±0.06) |
| 20-29 | 476 (773.7) | 186 (750.6) | 0.62 (±0.06) | 0.24 (±0.04) |
| 30-39 | 792 (987.7) | 318 (960.2) | 0.80 (±0.06) | 0.33 (±0.04) |
| 40-49 | 704 (1,102.1) | 264 (1,072.2) | 0.64 (±0.05) | 0.25 (±0.03) |
| 50-59 | 507 (999.4) | 183 (968.4) | 0.51 (±0.04) | 0.19 (±0.03) |
| 60-69 | 333 (814.7) | 68 (790.9) | 0.41 (±0.04) | 0.09 (±0.02) |
| 70-79 | 188 (581.4) | 32 (563.5) | 0.32 (±0.05) | 0.06 (±0.02) |
| 80-89 | 67 (313.1) | 9 (218.9) | 0.21 (±0.05) | 0.04 (±0.03) |
| 90-99 | ≤5 | ≤5 | | |
| *Geographical area* | | | | |
| North East | 53 (91.1) | 25 (91.2) | 0.58 (±0.16) | 0.27 (±0.11) |
| North West | 385 (648.3) | 155 (637.5) | 0.59 (±0.06) | 0.24 (±0.04) |
| Yorkshire & The Humber | 101 (173.8) | 43 (174.0) | 0.56 (±0.11) | 0.25 (±0.07) |
| East Midlands | 82 (186.2) | 25 (186.4) | 0.44 (±0.10) | 0.13 (±0.05) |
| West Midlands | 260 (542.8) | 98 (531.2) | 0.48 (±0.06) | 0.18 (±0.04) |
| East of England | 275 (472.3) | 92 (466.8) | 0.58 (±0.07) | 0.20 (±0.04) |
| South West | 269 (485.1) | 88 (478.0) | 0.55 (±0.07) | 0.18 (±0.04) |
| South Central | 307 (596.3) | 123 (586.6) | 0.51 (±0.06) | 0.21 (±0.04) |
| London | 271 (541.2) | 74 (524.2) | 0.50 (±0.06) | 0.14 (±0.03) |
| South East Coast | 322 (552.6) | 84 (533.7) | 0.58 (±0.06) | 0.16 (±0.03) |
| Northern Ireland | 149 (196.7) | 65 (186.5) | 0.76 (±0.12) | 0.35 (±0.08) |
| Scotland | 312 (598.1) | 92 (562.0) | 0.52 (±0.06) | 0.16 (±0.03) |
| Wales | 308 (656.2) | 107 (616.7) | 0.47 (±0.05) | 0.17 (±0.03) |

Note: results are not shown where the number of cases is ≤5

**Supplementary Table S7. Percentage prevalence of AS by calendar year and stratified by sex, age-group and geographical area (N = 7,532,980)**

| | Percentage prevalence (95% CI) | |
|---|---|---|
| | Main analysis | Sensitivity analysis |
| Overall | 0.15 (±0.003) | 0.06 (±0.002) |
| *Year* | | |
| 1998 | 0.13 (±0.006) | 0.07 (±0.004) |
| 1999 | 0.14 (±0.006) | 0.07 (±0.004) |
| 2000 | 0.14 (±0.005) | 0.07 (±0.004) |
| 2001 | 0.14 (±0.005) | 0.07 (±0.003) |
| 2002 | 0.15 (±0.005) | 0.07 (±0.003) |
| 2003 | 0.15 (±0.004) | 0.07 (±0.003) |
| 2004 | 0.16 (±0.004) | 0.07 (±0.003) |
| 2005 | 0.16 (±0.004) | 0.07 (±0.003) |
| 2006 | 0.16 (±0.004) | 0.07 (±0.003) |
| 2007 | 0.16 (±0.004) | 0.07 (±0.003) |
| 2008 | 0.17 (±0.004) | 0.07 (±0.003) |
| 2009 | 0.17 (±0.004) | 0.08 (±0.003) |
| 2010 | 0.17 (±0.004) | 0.08 (±0.003) |
| 2011 | 0.17 (±0.004) | 0.08 (±0.003) |
| 2012 | 0.17 (±0.004) | 0.08 (±0.003) |
| 2013 | 0.17 (±0.005) | 0.08 (±0.003) |
| 2014 | 0.17 (±0.005) | 0.08 (±0.003) |
| 2015 | 0.18 (±0.005) | 0.08 (±0.003) |
| 2016 | 0.18 (±0.006) | 0.08 (±0.003) |
| 2017 | 0.18 (±0.006) | 0.07 (±0.004) |
| *Sex* | | |
| Female | 0.08 (±0.003) | 0.03 (±0.002) |
| Male | 0.23 (±0.005) | 0.10 (±0.003) |
| *Age group* | | |
| 18-19 | 0.001 (±0.001) | 0.001 (±0.0003) |
| 20-29 | 0.03 (±0.001) | 0.016 (±0.001) |
| 30-39 | 0.11 (±0.002) | 0.06 (±0.002) |
| 40-49 | 0.18 (±0.003) | 0.09 (±0.002) |
| 50-59 | 0.23 (±0.003) | 0.11 (±0.002) |
| 60-69 | 0.26 (±0.003) | 0.11 (±0.002) |
| 70-79 | 0.21 (±0.004) | 0.07 (±0.002) |
| 80-89 | 0.14 (±0.004) | 0.03 (±0.002) |
| 90-99 | 0.09 (±0.007) | 0.01 (±0.003 |
| *Geographical area* | | |
| North East | 0.16 (±0.02) | 0.09 (±0.02) |
| North West | 0.17 (±0.01) | 0.07 (±0.01) |
| Yorkshire & The Humber | 0.15 (±0.02) | 0.07 (±0.01) |
| East Midlands | 0.15 (±0.02) | 0.06 (±0.01) |
| West Midlands | 0.14 (±0.01) | 0.06 (±0.01) |
| East of England | 0.16 (±0.01) | 0.07 (±0.01) |
| South West | 0.17 (±0.01) | 0.07 (±0.01) |
| South Central | 0.16 (±0.01) | 0.07 (±0.01) |
| London | 0.11 (±0.01) | 0.04 (±0.004) |
| South East Coast | 0.17 (±0.01) | 0.06 (±0.01) |
| Northern Ireland | 0.18 (±0.02) | 0.08 (±0.01) |
| Scotland | 0.17 (±0.01) | 0.05 (±0.01) |
| Wales | 0.15 (±0.01) | 0.06 (±0.01) |



Crossfield SSR, *et al. RMD Open* 2021; 7:e001888. doi: 10.1136/rmdopen-2021-001888

**Supplementary Figure S1. Person-years (million) in the incidence 'at-risk' cohort per year, 1998-2017 (N = 8,052,546)**

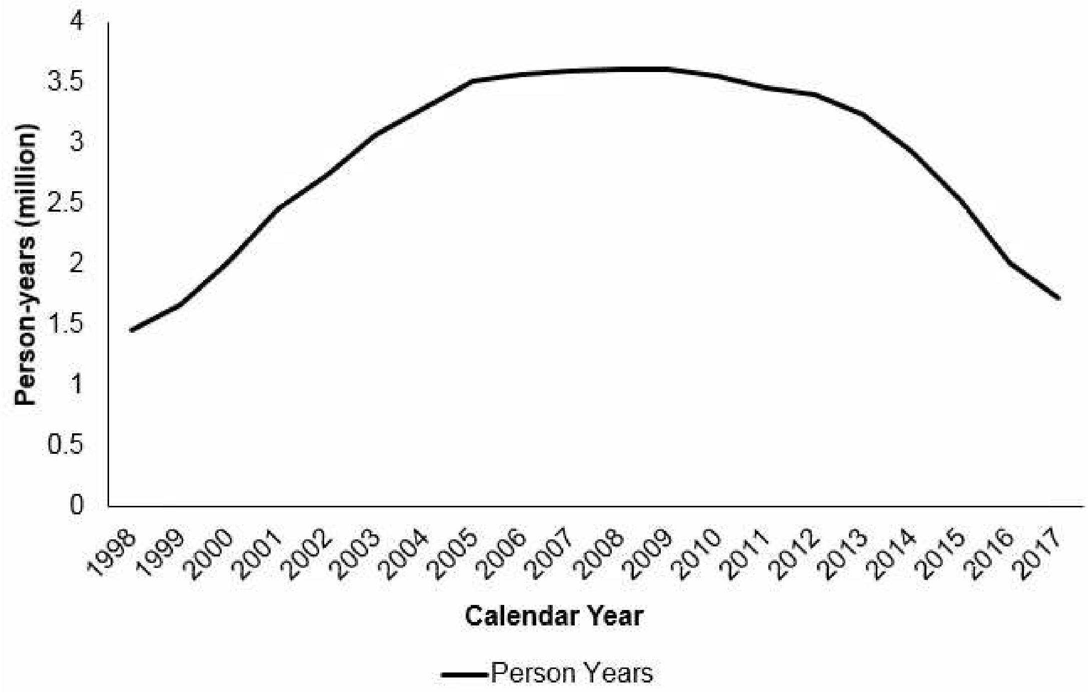


**Supplementary Figure S2. Annual incidence rate of AS defined as having ≥1 AS diagnostic code, 1998-2017 (N = 8,052,546), and in the sensitivity analysis, 1998-2016 (N = 7,919,770)**

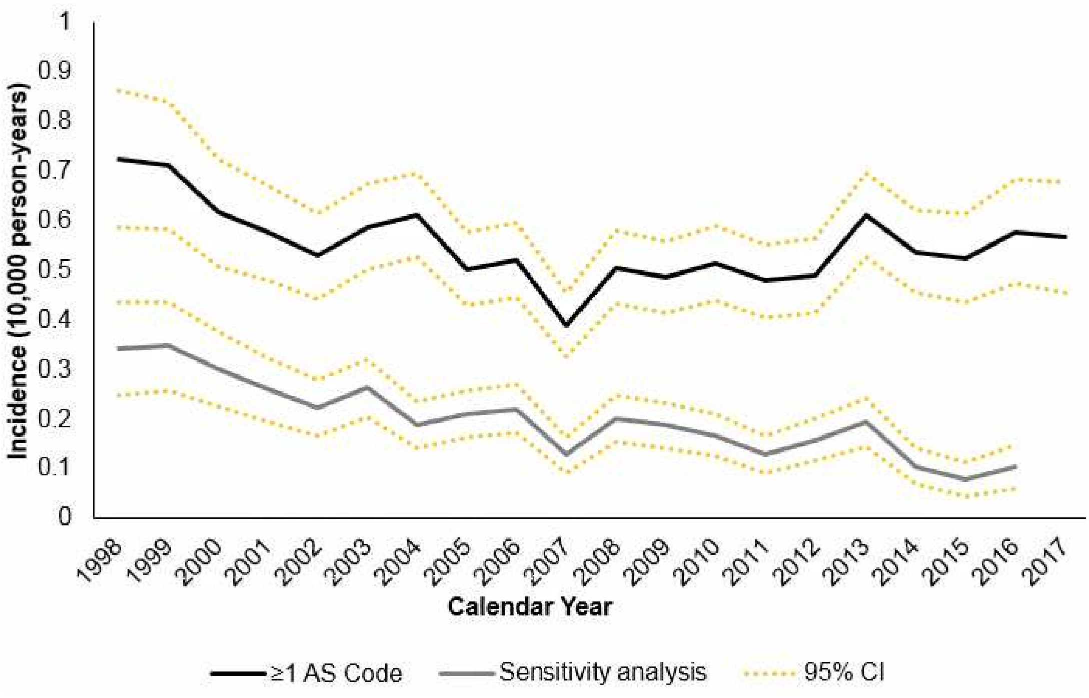

**Supplementary Figure S3. Annual incidence rate of AS in women and men, 1998-2017 (N = 8,052,546)**

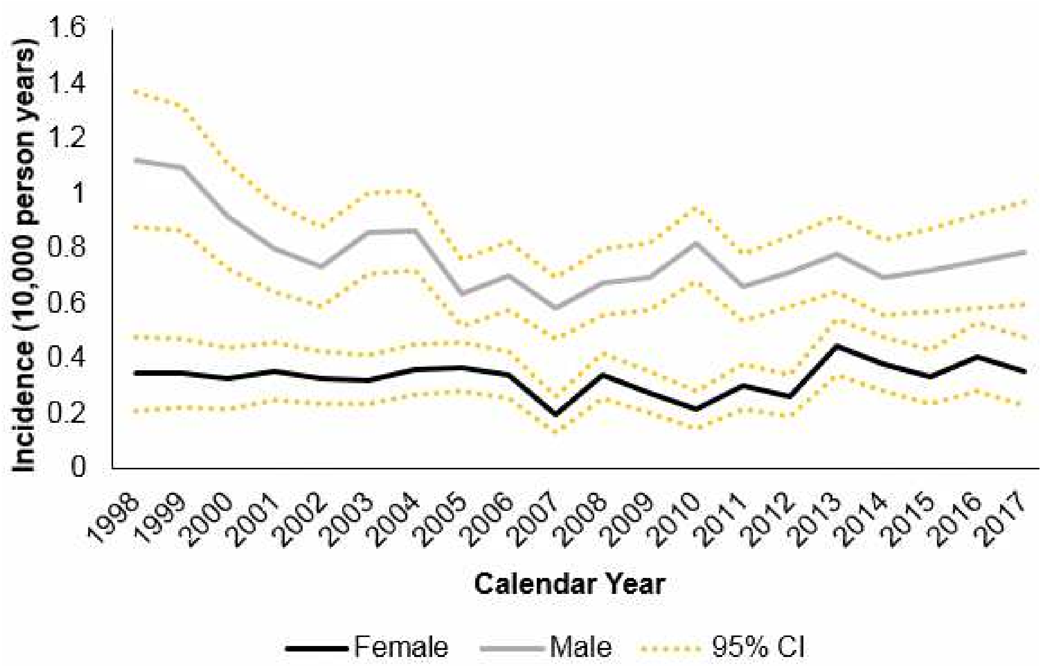


**Supplementary Figure S4. Annual incidence rate of AS by age-group, 1998-2017 (N = 8,051,097)**

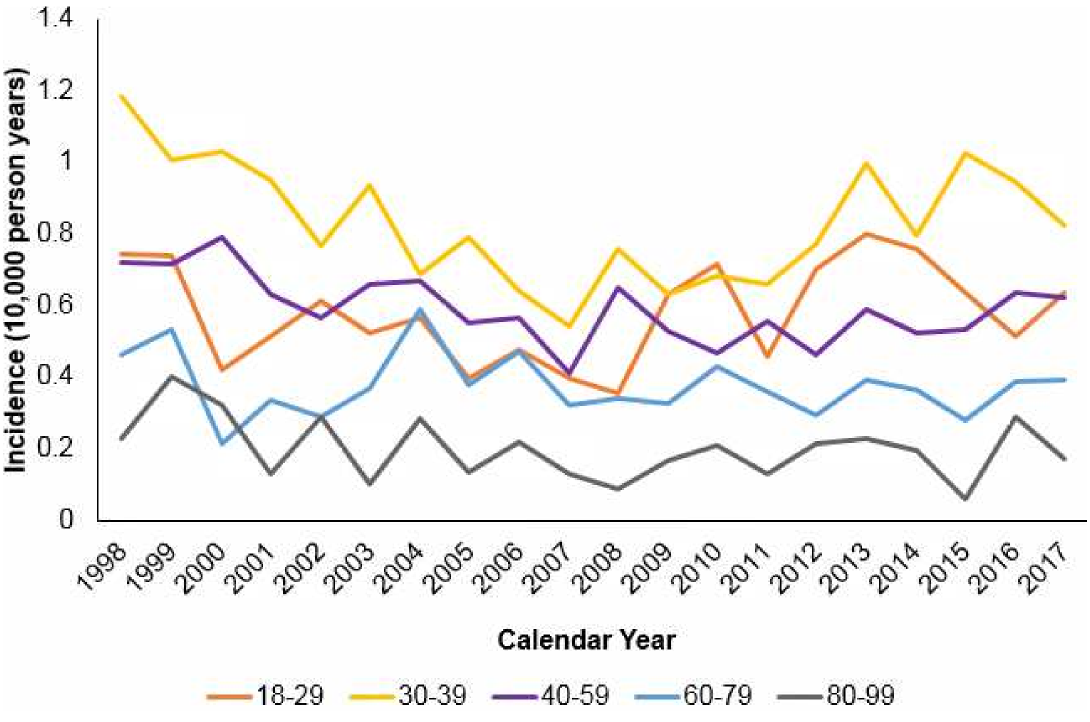




Crossfield SSR, *et al. RMD Open* 2021; 7:e001888. doi: 10.1136/rmdopen-2021-001888

**Supplementary Figure S5. Annual incidence rate of AS by geographic region, 1998-2017 (N = 8,044,388)**

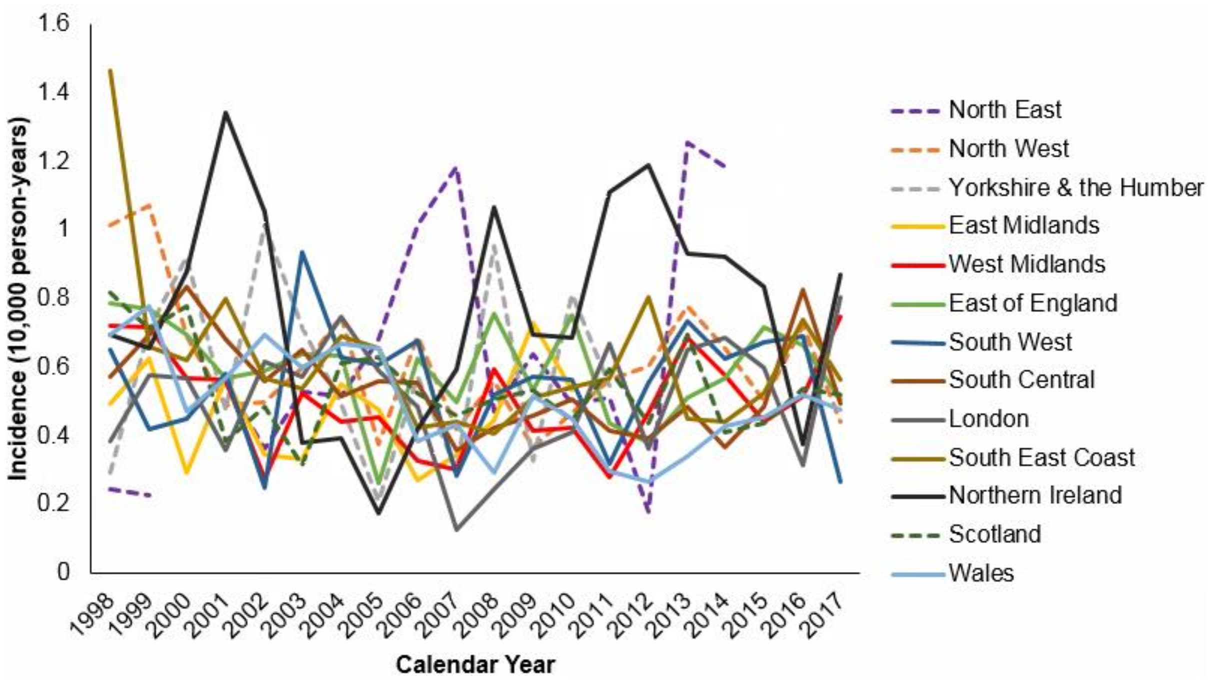


**Supplementary Figure S6. Annual incidence rate of AS in women and men in the sensitivity analysis, 1998-2017 (N = 7,919,770)**

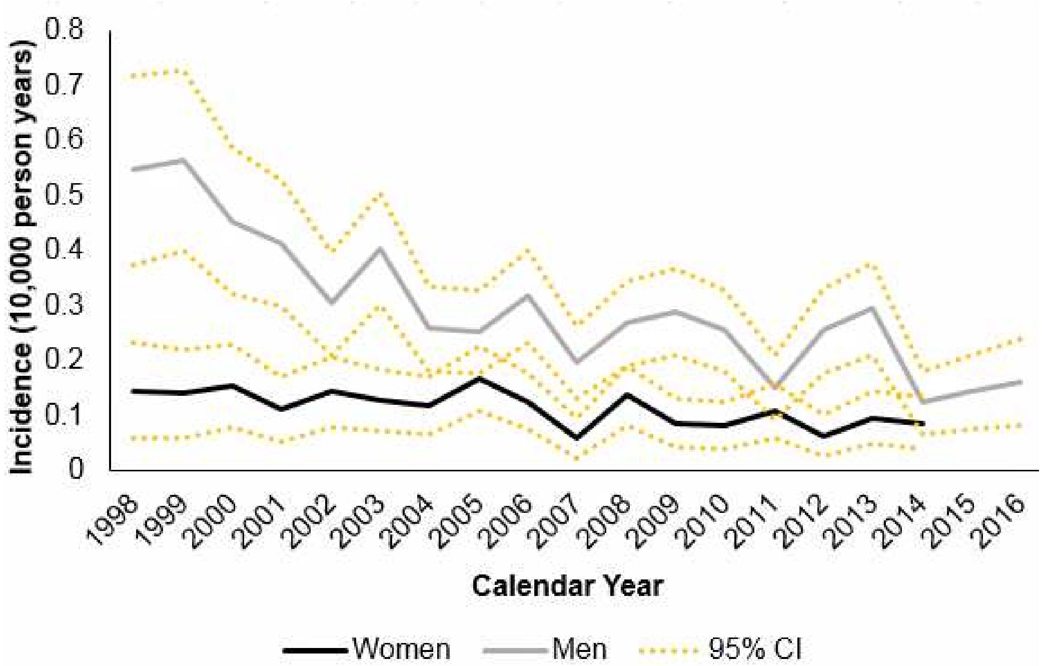

**Supplementary Figure S7. Annual incidence rate of AS in the sensitivity analysis by age-group, 1998-2017 (N = 7,918,339)**

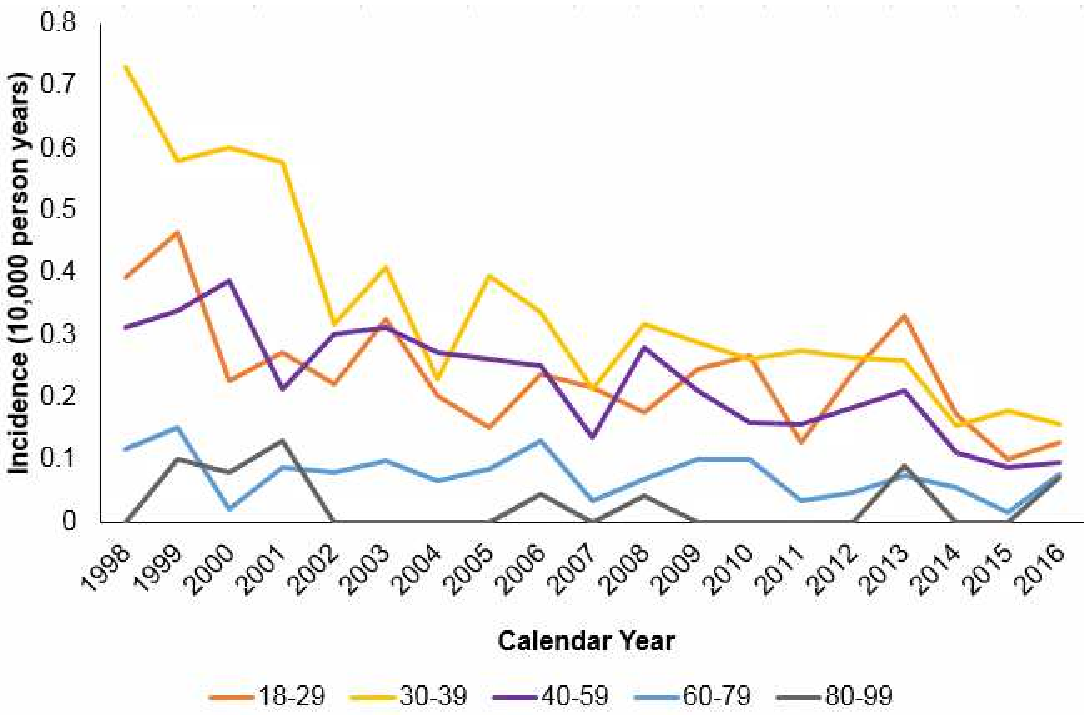


**Supplementary Figure S8. Annual percentage prevalence of AS per age-group among patients aged 18-99 in the sensitivity analysis, 1997-2016 (N = 7,506,959)**

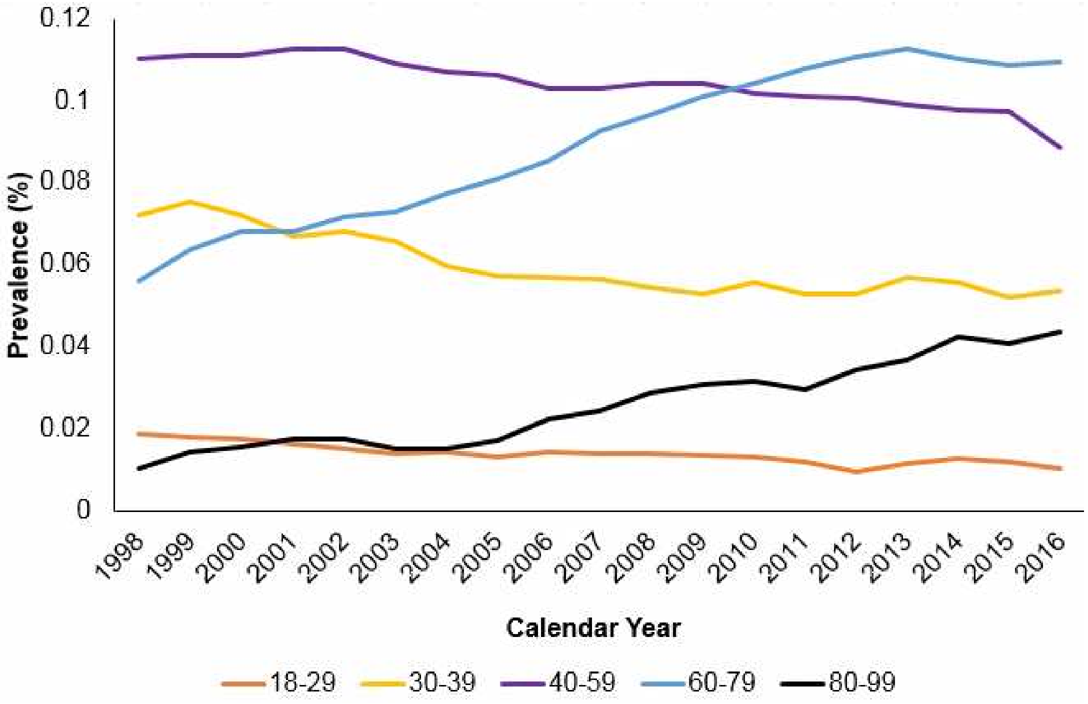

**Supplementary Figure S9. Annual percentage of patients diagnosed with AS (having ≥1, ≥2 and ≥3 years of prior UTS registration) that had a prior back-pain symptom code, 1998-2017 (N = 3,101; N = 2,734; N = 2,417)**

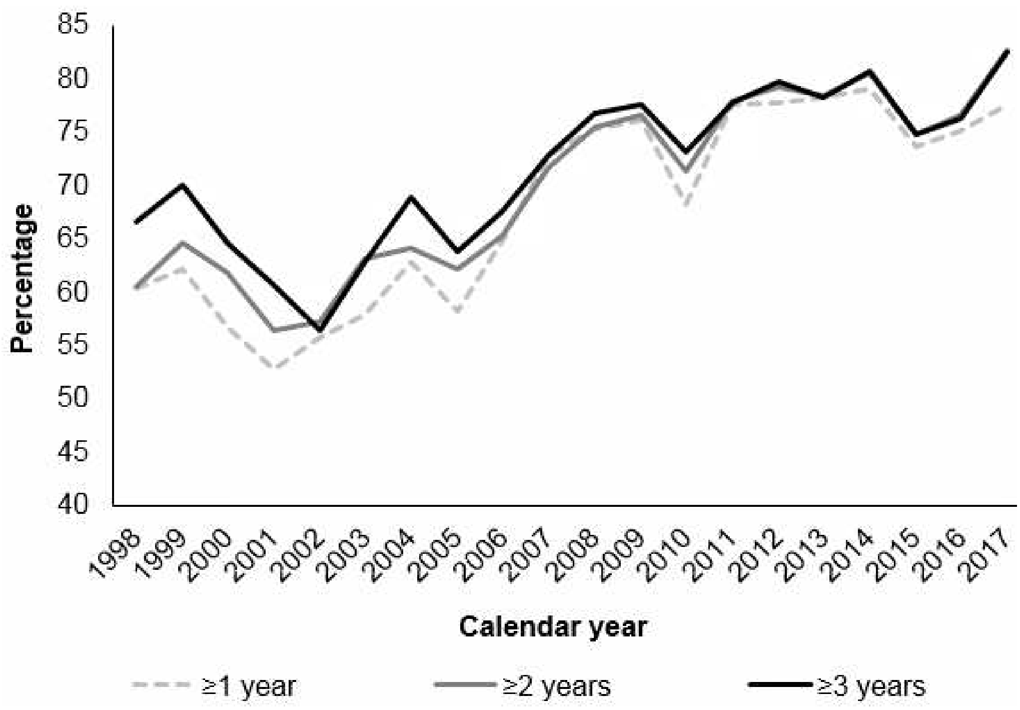


**Supplementary Figure S10. Annual percentage of patients diagnosed with AS (having ≥1, ≥2 and ≥3 prior years of UTS) that had a prior back-pain symptom code, for women and men 1998-2017 (N = 3,101; N = 2,734; N = 2,417)**

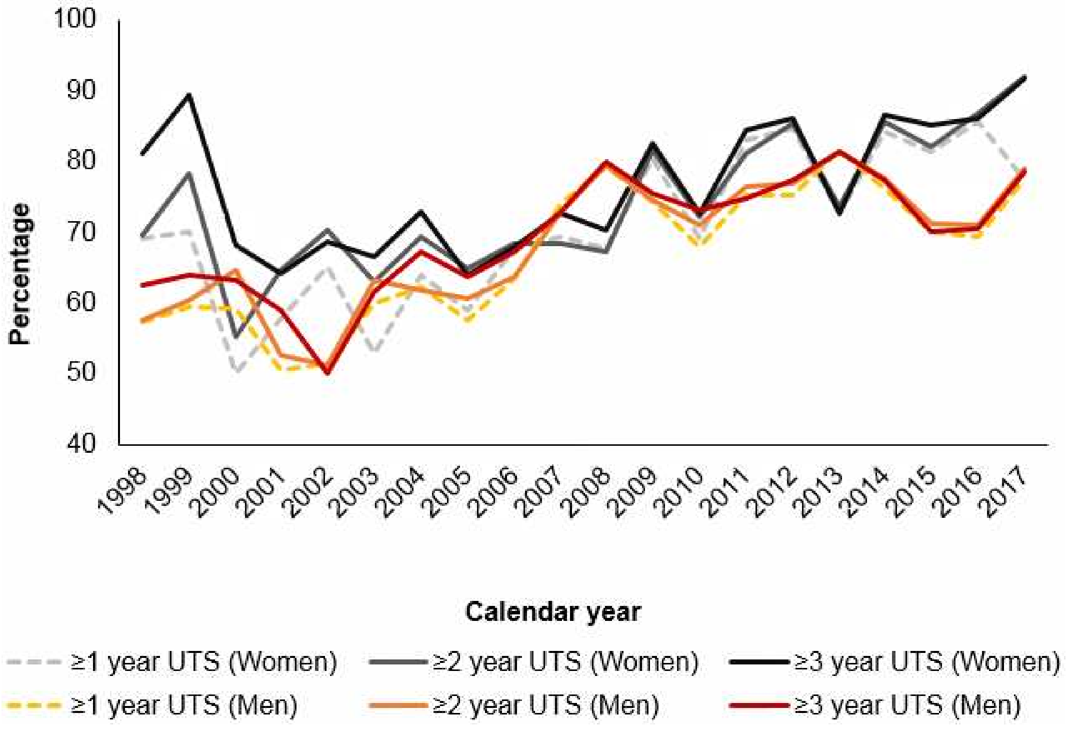

**Supplementary Figure S11. Annual percentage of patients with in the sensitivity analysis, having ≥1, ≥2 and ≥3 prior years of UTS, who had a prior back-pain symptom code, 1998-2016 (N = 1,071; N = 957; N = 821)**

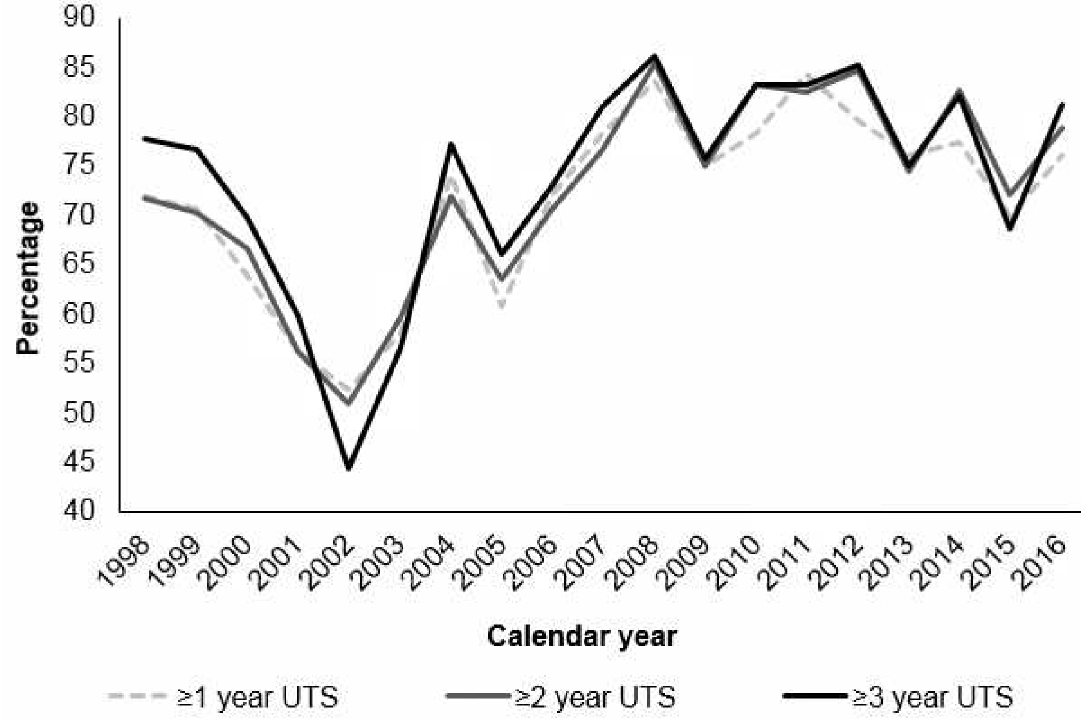




Crossfield SSR, *et al. RMD Open* 2021; 7:e001888. doi: 10.1136/rmdopen-2021-001888

**Supplementary Figure S12. Annual percentage of patients in the sensitivity analysis, having ≥1, ≥2 and ≥3 prior years of UTS, who had a prior back-pain symptom code, for women and men, 1998-2016 (N = 1,071; N = 957; N = 821)**

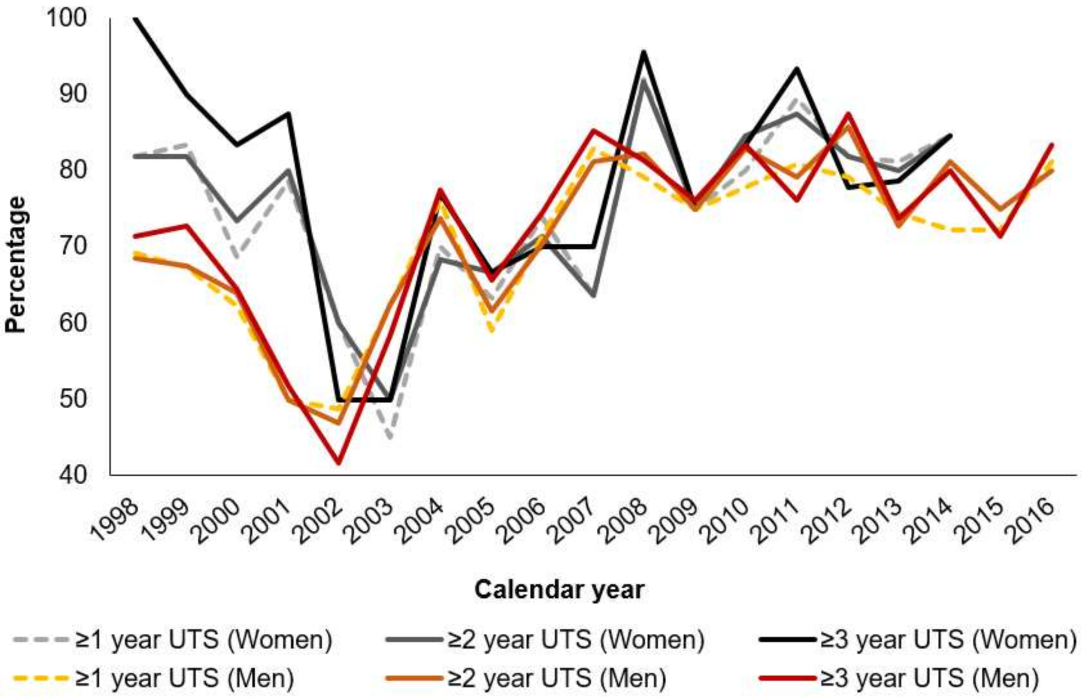


Note: data suppressed where there are ≤5 cases

**Supplementary Figure S13. Median time in years from first recorded symptom to first diagnosis in the sensitivity analysis, 1998-2016 (N = 757)**

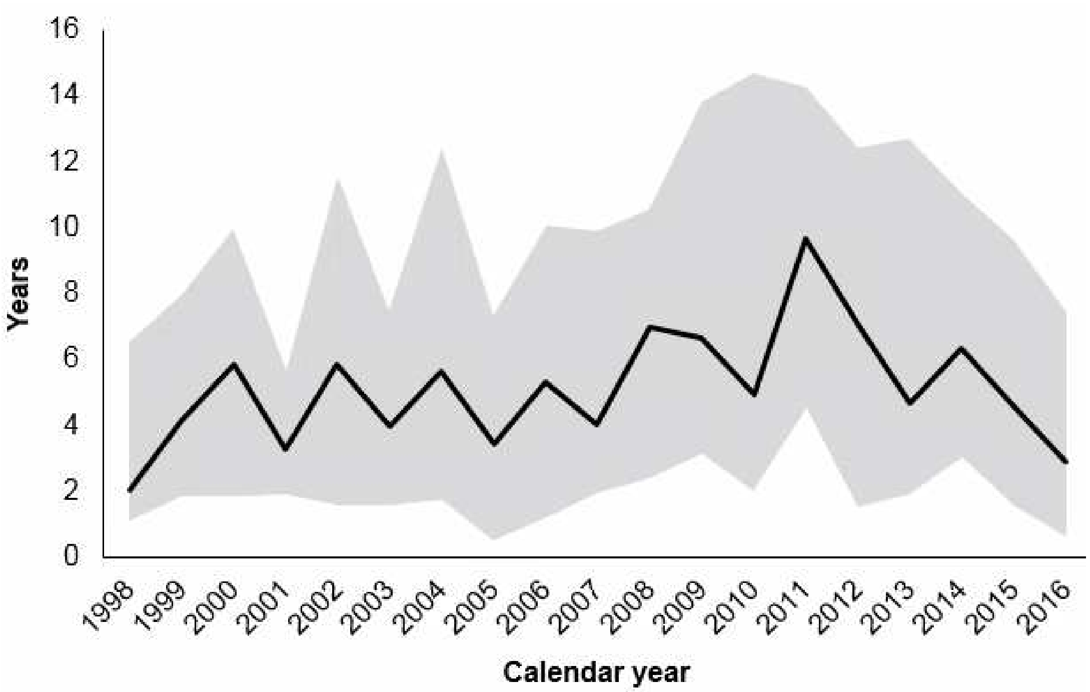


**Supplementary Figure S14. Median time in years from first recorded symptom to first diagnosis in the sensitivity analysis, for women and men, 1998-2016 (N = 757)**

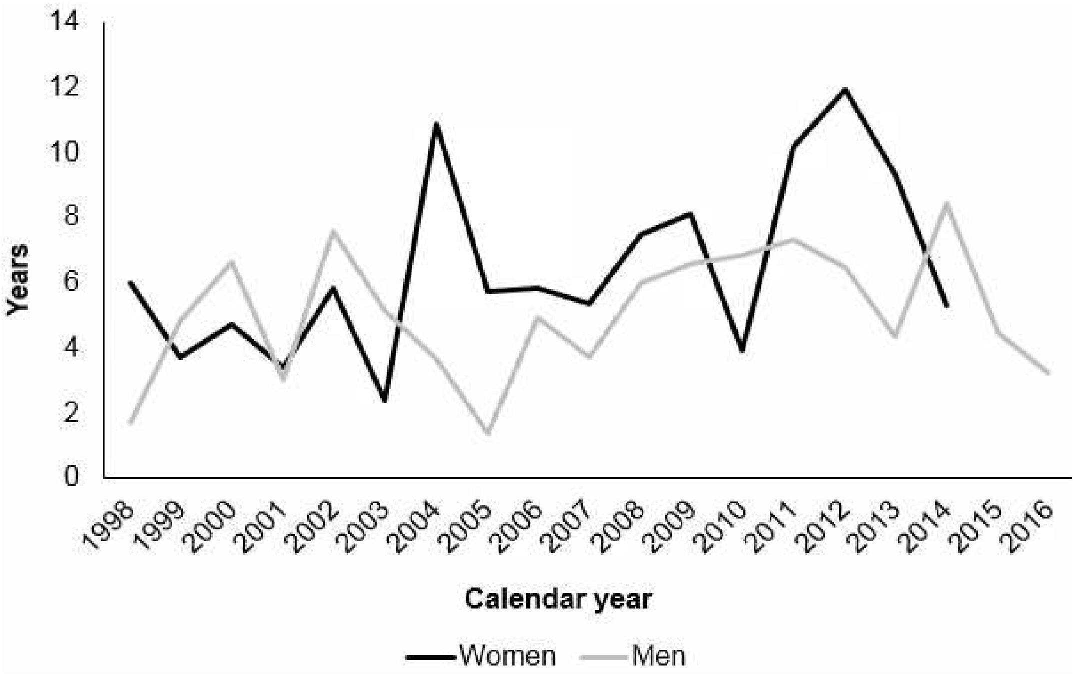


Note: data suppressed where there are ≤5 cases



Crossfield SSR, *et al. RMD Open* 2021; 7:e001888. doi: 10.1136/rmdopen-2021-001888

**Supplementary Figure S15. Median time in years from symptom to diagnosis, for patients with ≥1, ≥2 and ≥3 years prior UTS registration, 1998-2017 (N = 2,120; N = 1,929; N = 1,750)**

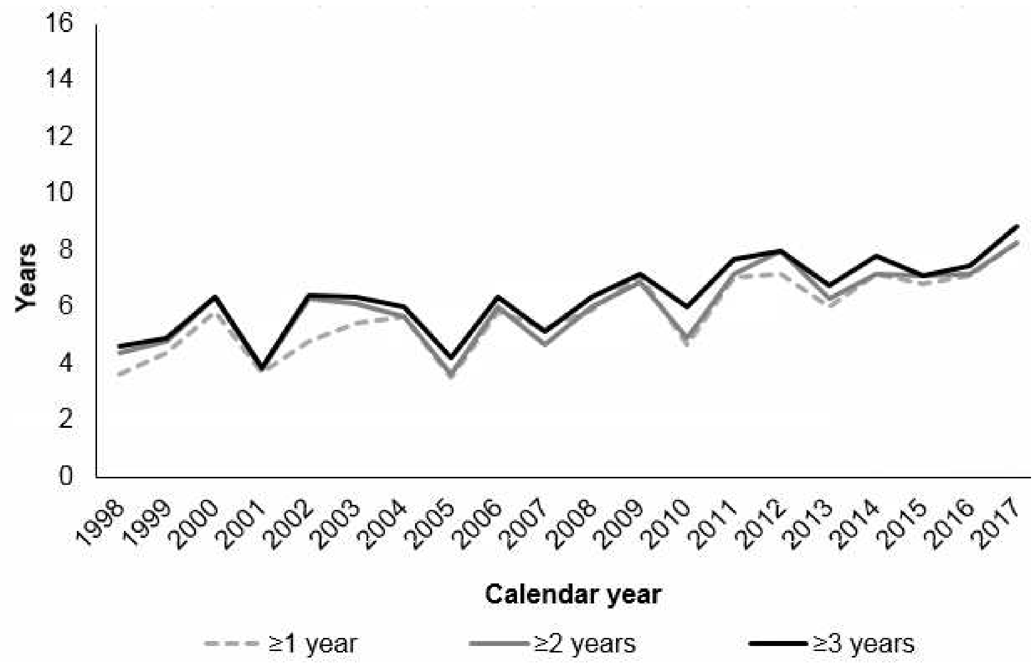


**Supplementary Figure S16. Median time in years from symptom to diagnosis, for patients with ≥1, ≥2 and ≥3 years prior UTS registration in the sensitivity analysis, 1998-2016 (N = 757; N = 688; N = 606)**

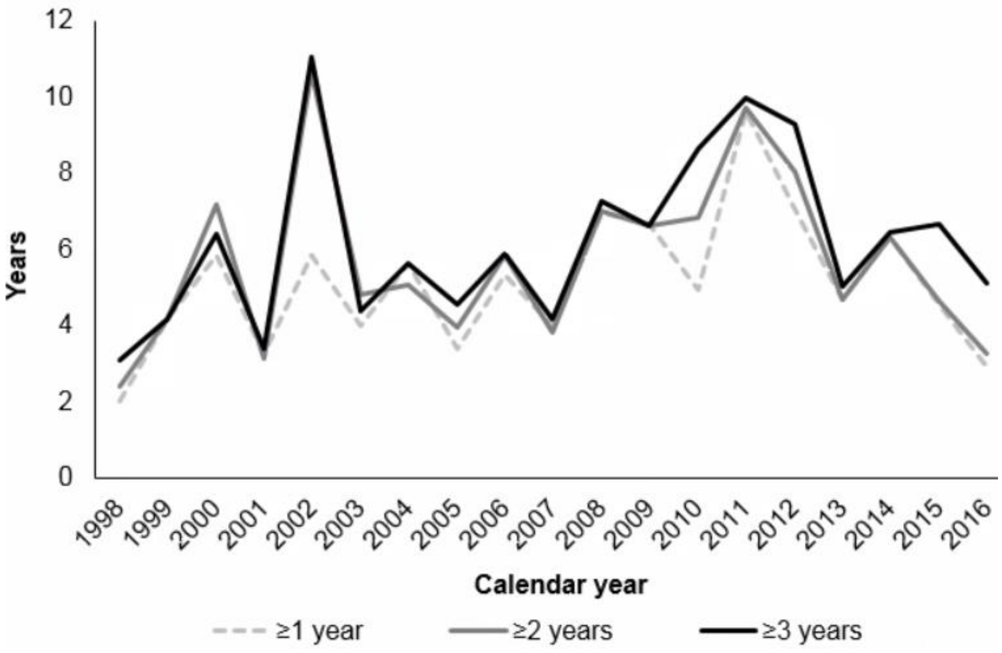

**Supplementary Figure S17. Annual percentage of patients diagnosed with AS that had prior back pain and rheumatology referral, for women and men 1998-2017 (N = 3,101)**

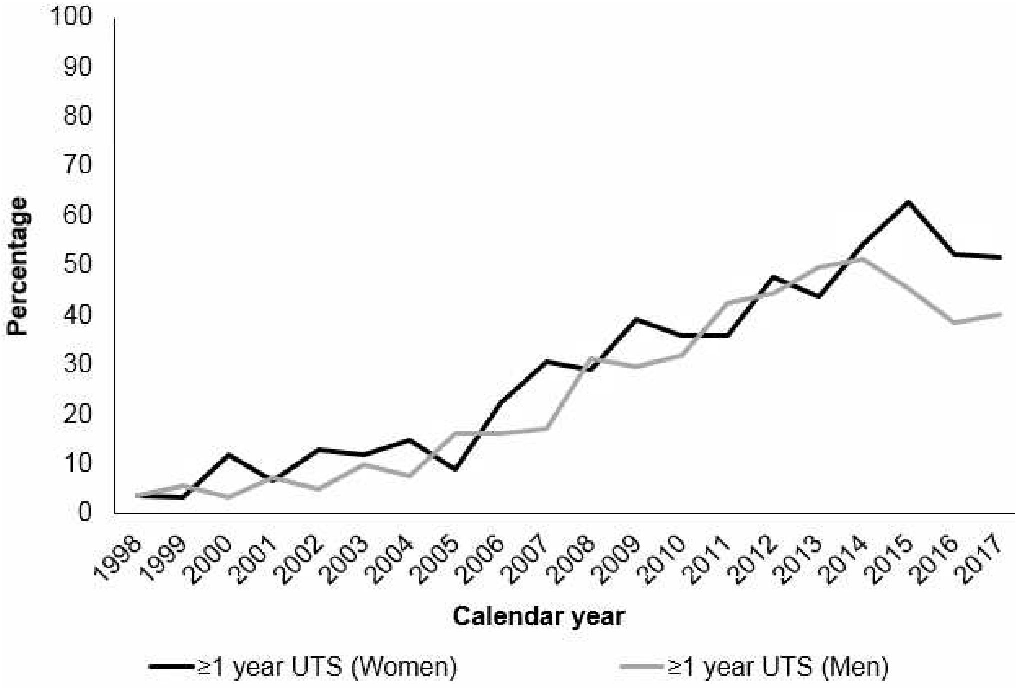


**Supplementary Figure S18. Annual percentage of patients diagnosed with AS that had a prior rheumatology referral, for women and men 1998-2017 (N = 3,101)**

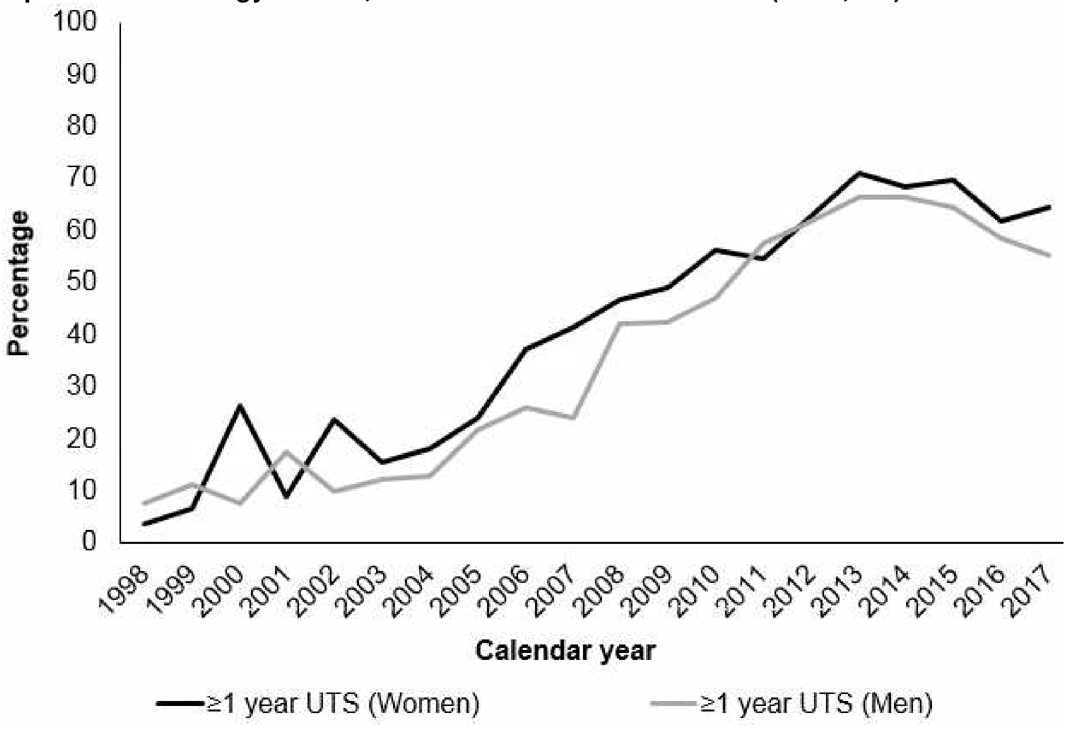

**Supplementary Figure S19. Annual median time in years from first recorded back pain to rheumatology referral, for men and women, 1998-2017 (N = 819)**

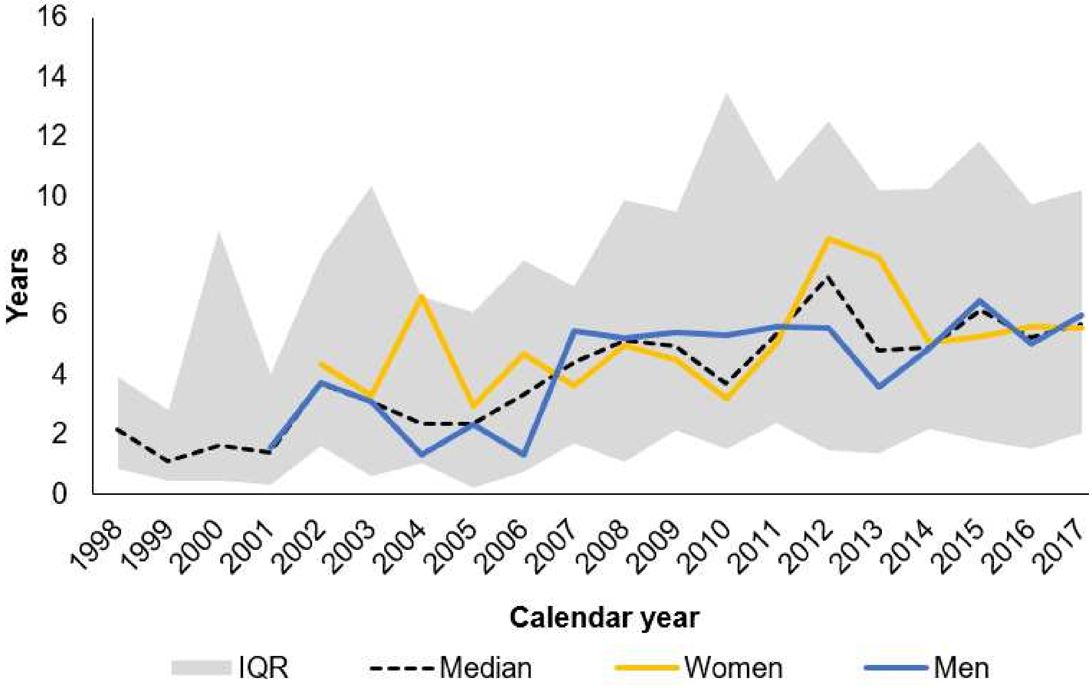


Note: data suppressed where there are ≤5 cases



Crossfield SSR, *et al. RMD Open* 2021; 7:e001888. doi: 10.1136/rmdopen-2021-001888

**Supplementary Figure S20. Annual median time in years from first rheumatology referral to diagnosis, for women and men: A) primary analysis, 1998-2017 (N = 1,167); B: sensitivity analysis, 1998-2016 (N = 399)**

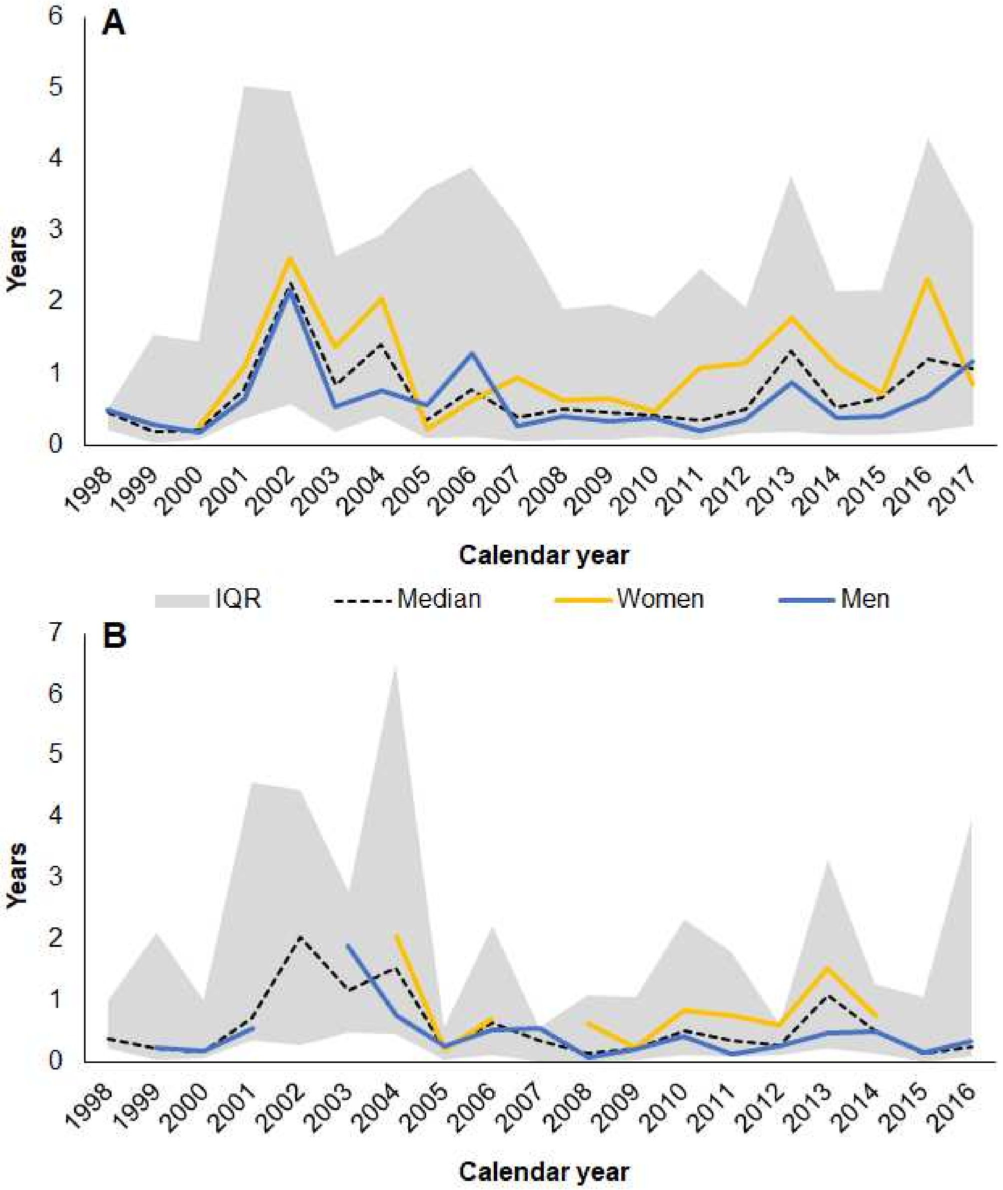


Note: data suppressed where there are ≤5 cases



Crossfield SSR, *et al. RMD Open* 2021; 7:e001888. doi: 10.1136/rmdopen-2021-001888

**Supplementary Figure S21. Annual median time in years from first recorded back pain symptom to rheumatology referral, for men and women in the sensitivity analysis, 1998-2016 (N = 279)**

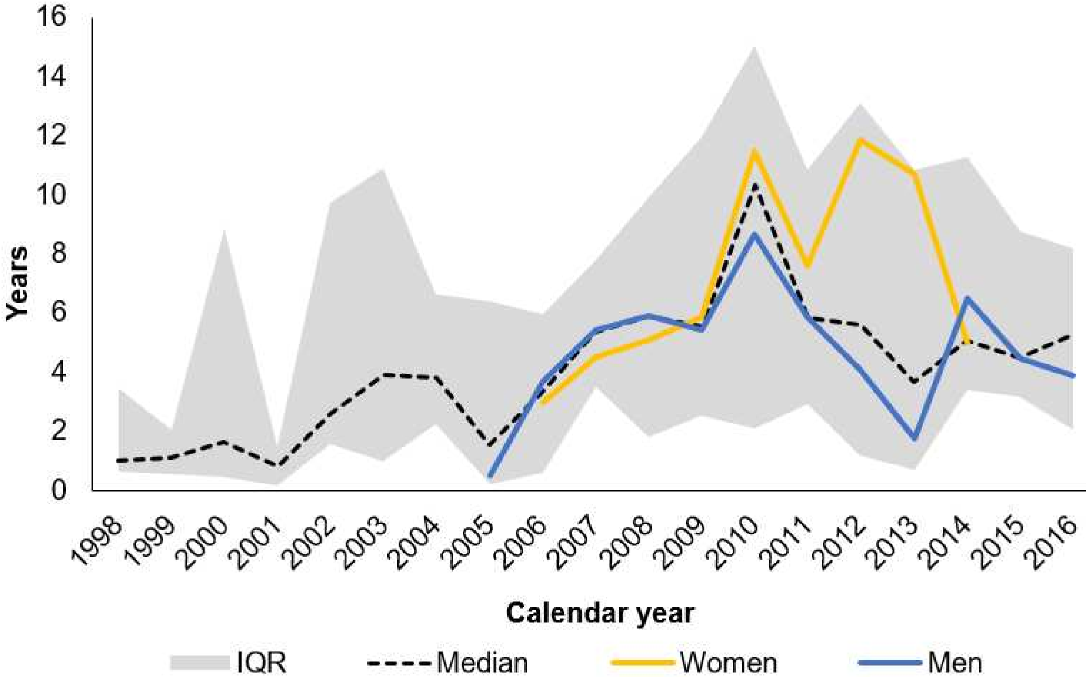


Note: data suppressed where there are ≤5 cases



Crossfield SSR, *et al. RMD Open* 2021; 7:e001888. doi: 10.1136/rmdopen-2021-001888
